# Oral Delivery Systems for Food‐Derived Bioactive Peptides: Enhancing Stability, Bioavailability, and Health Benefits

**DOI:** 10.1002/advs.76666

**Published:** 2026-07-23

**Authors:** Yu Xu, Fan Li, Runan Zhao, Tao Shu, Weihong Min, Bei‐Wei Zhu

**Affiliations:** ^1^ State Key Laboratory For Development and Utilization of Forest Food Resources Zhejiang A&F University Hangzhou P. R. China; ^2^ School of Health Science and Engineering University of Shanghai for Science and Technology Shanghai P. R. China; ^3^ College of Food and Health Zhejiang A&F University Hangzhou P. R. China; ^4^ SKL of Marine Food Processing and Safety Control National Engineering Research Center of Seafood Collaborative Innovation Center of Seafood Deep Processing School of Food Science and Technology Dalian Polytechnic University Dalian P. R. China; ^5^ College of Biosystems Engineering and Food Science Fuli Institute of Food Science Zhejiang University Hangzhou P. R. China; ^6^ School of Agriculture Food and Ecosystem Sciences Faculty of Science The University of Melbourne Parkville VIC Australia

**Keywords:** bioavailability, chronic diseases, delivery systems, food‐derived bioactive peptides, oral delivery

## Abstract

Food‐derived bioactive peptides (FBPs) represent a promising class of nutraceuticals for the prevention and management of chronic diseases due to their diverse bioactivities and high biocompatibility; however, their therapeutic potential is severely limited by low oral bioavailability resulting from instability during processing, sensory drawbacks such as bitterness, and extensive gastrointestinal degradation. This review systematically outlines the major bottlenecks hindering the oral delivery of FBPs, including sensitivity to environmental factors (temperature, pH, metal ions) and physiological barriers (enzymatic hydrolysis, mucus layer, and epithelial transport). To overcome these challenges, advanced delivery systems, such as nanoparticles (NPs), nanoemulsions (NEs), liposomes, hydrogels, and composite carriers, have been designed. These engineered systems have demonstrated enhanced bioactivity and targeted delivery in preclinical models of neuroprotection, anti‐inflammatory therapy, metabolic regulation, and cancer treatment, often outperforming free peptides in terms of stability and intestinal absorption. However, establishing quantitative correlations between formulation parameters and in vivo peptide bioavailability remains a critical bottleneck. Future research should focus on rationally designed stimuli‐responsive platforms grounded in intestinal physiology, supported by standardized frameworks for scalable manufacturing and safety evaluation. This work provides a comprehensive framework for advancing FBPs delivery platforms toward functional food and chronic disease management applications.

## Graphical abstract

Advanced delivery systems—including nanoparticles, nanoemulsions, liposomes, and hydrogels—protect food‐derived bioactive peptides from gastrointestinal degradation and bitterness. These systems enable pH‐responsive release, enhance intestinal absorption, and improve therapeutic efficacy against oxidative stress, metabolic disorders, cancer, and neurodegeneration, thereby unlocking the full potential of FBPs for chronic disease management.

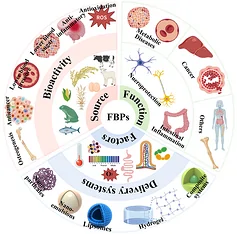

## 1 Introduction

Food‐derived bioactive peptides (FBPs) have shown potential in the adjuvant treatment and symptom management of chronic diseases due to their high biological safety and multiple bioactivities. These peptides, usually derived from the enzymatic hydrolysis, fermentation, or hydrolysis of food proteins from animals, plants, and marine sources, are short‐chain peptides composed of 2–20 amino acids. While retaining nutritional value, they demonstrate physiological functions including antihypertensive, anti‐inflammatory, and anti‐tumor activities owing to their specific amino acid sequences. For instance, whey protein peptides can exert effects by enhancing the activity of acetylcholinesterase (AChE) [1]; plant‐derived peptides can effectively counteract excessive inflammatory responses by regulating inflammatory signaling pathways and inhibiting the secretion of pro‐inflammatory cytokines [2]; tuna‐derived peptides have been shown to alleviate chemotherapy‐induced immunosuppression and intestinal damage, along with potential anti‐tumor activities [3]. Compared with chemically synthesized drugs, natural bioactive peptides possess superior safety and lower side effects, which aligns with the demands for chronic disease prevention and health promotion [4]. In addition, studies have demonstrated that regular intake of FBPs can yield health benefits by improving disease‐related biomarkers, providing a safe and effective alternative for the prevention and treatment of various chronic diseases [5].

Although FBPs possess diverse bioactivities, their biological activities can be significantly influenced by factors such as source, temperature, pH, and metal ions during production, processing, storage, and transportation. Moreover, oral administration of FBPs faces multiple obstacles including bitterness, gastrointestinal digestion and absorption barriers, which ultimately restrict their bioavailability. Peptides from different protein sources exhibit varying bioactivities. Shu et al. found that under the same enzymatic hydrolysis conditions, the angiotensin‐converting enzyme (ACE) inhibitory activity of goat milk peptides (49.8%) was significantly higher than that of cow milk peptides (35.1%) [6]. Furthermore, production conditions, storage, and transportation have a profound impact on peptide bioactivity; for example, temperature, Ph, and metal ions all directly influence biological activities. Studies have indicated that the ACE inhibitory activity of tomato peptides is sensitive to environmental conditions, decreasing significantly at high temperatures (e.g., 100 °C), reaching its maximum under neutral pH, and declining under acidic or alkaline conditions. Likewise, the presence of metal ions such as Fe^2^^+^ and Cu^2^^+^ can markedly reduce ACE inhibitory activity by disrupting peptide chemical bonds, reducing solubility, and exposing hydrophobic groups [7]. Furthermore, direct oral administration first requires overcoming the bitterness of peptides [8]. Subsequently, upon entering the gastrointestinal tract, gastric acid (pH 1–3) can cause peptide bond cleavage; trypsin and carboxypeptidase exert enzymatic hydrolysis effects; and intestinal flora metabolism modifies peptide structures. In addition, the barriers formed by tight junctions between intestinal epithelial cells, mucus layer thickness, and unstirred water layer significantly limit the transmembrane transport efficiency of peptides, thereby reducing their bioavailability substantially [9]. Therefore, it is particularly crucial to develop technical systems that can protect bioactive peptides and improve their delivery efficiency.

Delivery systems represent a key technology to break through the bottleneck of FBPs bioavailability. Current research mainly focuses on various delivery systems including nanoparticles, nanoemulsions, liposomes, hydrogels and composite systems, and their application in managing conditions such as neuroprotection, enteritis, metabolic disorders and cancer. Among these systems, nanoparticles can effectively cross multiple barriers and promote peptide absorption by virtue of their nanoscale size [10]. For example, Youssef et al. encapsulated specific decoy peptides (DP) in polymeric chitosan nanoparticles (CSNP), which significantly reversed cognitive impairment in a mouse model of neuroinflammation [11]. In addition to addressing issues such as poor stability, oxidation and absorption of bioactive substances, emulsion systems can enhance the gastrointestinal stability of peptides, improve intestinal permeability and control release rates, thereby increasing bioavailability [12]. The unique bilayer structure of liposomes endows them with excellent biocompatibility, enabling them to fuse with cell membranes easily and enter cells, promote peptide distribution in tissues and organs, and prevent enzymatic degradation. Moreover, their self‐assembly ability and amenability to modification are highly desirable features [13, 14]. Liu et al. precisely encapsulated two peptides derived from egg white protein (RADHPFL and YAEERYPIL) into targeted liposomes. This encapsulation strategy significantly improved the stability of the peptides in the complex gastrointestinal environment, while fully preserving their ACE inhibitory activity, ensuring that they can still exert potent biological functions when reaching target sites [15]. Hydrogels possess biocompatibility and biodegradability, and their three‐dimensional network structure enables sustained and pH‐responsive release of peptides, which is compatible with the absorption characteristics of different regions in the gastrointestinal tract [16, 17]. Liu et al. constructed a pH‐responsive network using chitosan‐alginate hydrogels, which encapsulated and protected rice bran bioactive peptides (RBAP) in the acidic gastric environment, and achieved sustained release of over 70% of the peptides in the neutral intestinal environment. This system enhanced the free radical scavenging capacity by 3–7 times, protected cells from oxidative stress damage, and addressed the problem of low bioavailability [18]. In addition, composite delivery systems further optimize the stability and targeted delivery efficiency of FBPs by integrating the properties of multiple materials, providing innovative solutions for improving bioavailability. Wu et al. used sodium alginate‐coated liposomes to deliver dipeptidyl peptidase‐IV (DPP‐IV) inhibitory collagen peptides; the sodium alginate‐coated carriers exhibited significantly enhanced storage stability, gastrointestinal stability and transcellular permeability [19]. In summary, these systems maintain the structural integrity of peptides through physical encapsulation, barrier protection and controlled release mechanisms, enhance their mucosal penetration and intestinal absorption, prolong in vivo circulation, preserve peptide structures and improve bioavailability [20]. This review aims to clarify the bioactivities of FBPs, the bottlenecks limiting their oral utilization, and how to improve their bioavailability through rational design of delivery systems, thereby promoting the practical application of these bioactive components in chronic disease prevention, treatment and health promotion.

## 2 Sources and Bioactivities of FBPs

**FIGURE 1:**
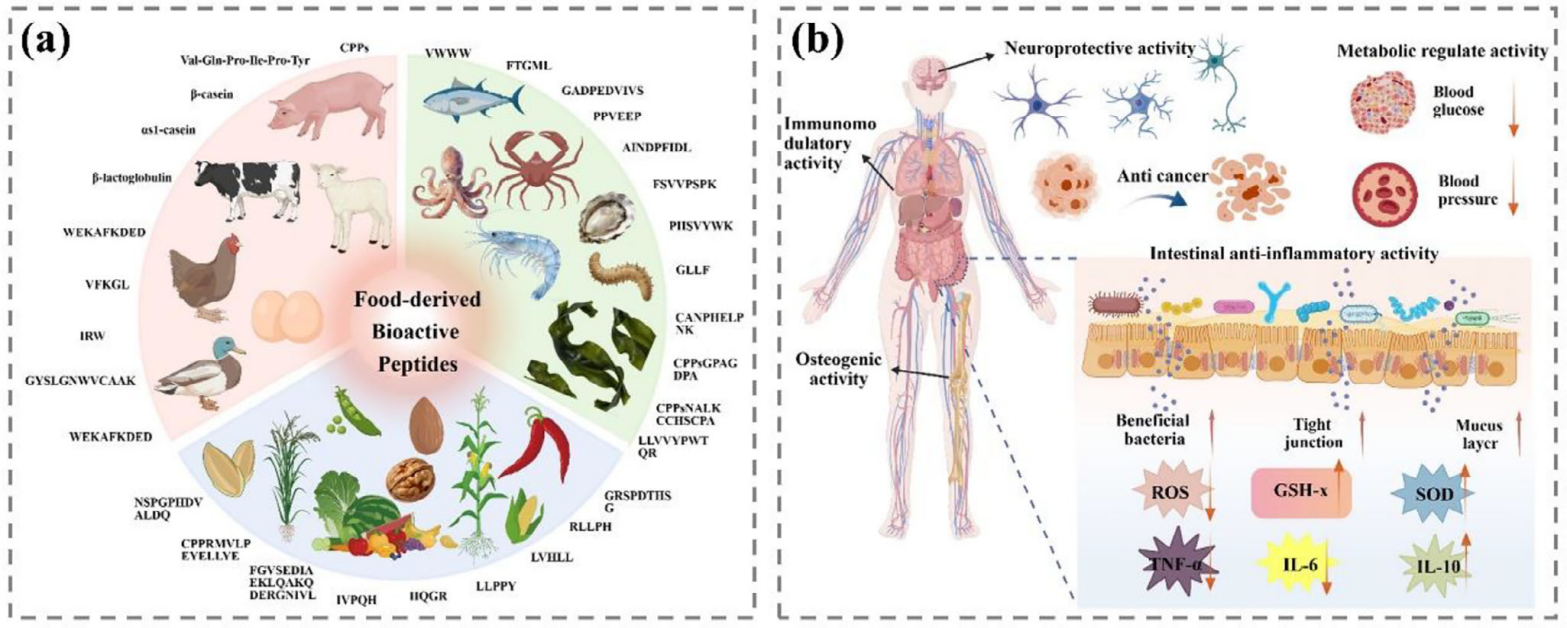
Sources and bioactivity of FBPs. (a) Source classification of FBPs. (b) Bioactivities of FBPs. Created with BioGDP.com [147].

FBPs refer to food‐derived peptidic compounds that are formed by the dehydration condensation of fewer than 100 amino acids, with a relative molecular mass below 6 kDa, and possess multiple biological activities including neuroprotection, anti‐inflammation, hypoglycemia, hypotension, and anti‐cancer effects [21]. They are mainly derived from the enzymatic hydrolysis of foodstuffs from animal, plant, and marine sources, and their bioactivities vary significantly depending on the origins (Figure 1a). Among these, animal‐derived peptides exhibit prominent efficacy in hypotension, antioxidation, and neuroprotection [22]. For instance, porcine‐derived peptides demonstrate excellent ACE inhibitory activity [23]; bovine blood peptides and duck embryo peptides possess strong free radical scavenging capacity [24, 25]; whey protein peptides can ameliorate neurological functions through synergistic mechanisms [1]. Plant‐derived peptides exert multi‐dimensional health benefits such as anti‐inflammation and antioxidation, and also demonstrate neuroprotective effects by regulating pathways like the renin‐angiotensin system [26]. Studies have shown that plant‐derived peptides can effectively counteract excessive inflammatory responses by modulating inflammatory signaling pathways and inhibiting the secretion of pro‐inflammatory cytokines [2]. Wang et al. determined the compositions of total amino acids and free amino acids of walnut peptide WPH, demonstrating that WPH is an effective free radical scavenger [27]. In addition, Song et al. found that hazelnut‐derived peptide YYLLVR upregulated the expression of proteins related to the ACE2/MAS axis by regulating the renin‐angiotensin system (RAS), and promoted the expression of BDNF, SYN‐1, and PSD95 in an Ang II‐induced neural injury model, thereby achieving neuronal protection [28]. Marine‐derived peptides feature diverse structures and exhibit unique potential in anti‐cancer, immune regulation, and memory impairment amelioration. For example, phycobiliprotein‐derived peptides from red algae display anti‐cancer, immunomodulatory, and neuroprotective properties [29]. Wang et al. identified candidate peptides such as VSLNLPYSVVRGEQFVVQA from the buccal glands of lampreys with anti‐inflammatory, antibacterial, and ACE inhibitory activities through peptidomics analysis, proving that marine‐derived peptides have great development potential [30]. Zhao et al. discovered that tuna hydrolysate could alleviate 5‐FU‐induced immunosuppression and intestinal mucosal damage, and had potential anti‐tumor effects [3]. Moreover, emerging protein sources also contribute peptides with antioxidant, ACE inhibitory, and metabolic regulatory activities [31]. Among them, insect bioactive peptides (IBPs) have been confirmed to possess antioxidant, free radical scavenging, and ACE inhibitory properties through in vitro and in vivo studies, and their anti‐diabetic, anti‐obesity, and anti‐inflammatory activities have also been widely reported [32]. In conclusion, FBPs from different sources have distinct targets of action and physiological effects (Table 1). These peptides not only have diverse biological activities, but also possess good biocompatibility due to their natural origin, thus holding important application value in the prevention and adjuvant treatment of chronic diseases (Figure 1b).

**TABLE 1:** Summary of bioactive peptides from different food sources and their major activities.

Source	Type	Peptide sequence/ Hydrolysate	Major Bioactivity	Mechanism of action	References
animal	Pork liver and placenta	FWG, MFLG, SDPPLVFVG and FFNDA	Lower blood pressure	ACE inhibitory activity	[23]
	cow blood	GFAGDDAPRA, SGPGIHVR, APSADAPM, TQRFFESF and HVDPENFKLL	Antioxidation	Free radical scavenging capacity; enhances the body's antioxidant capacity by activating the cellular Keap1‐Nrf2 signaling pathway	[24]
	Duck embryo	TVDGPSGKLWRD	Antioxidation	Free radical scavenging capacity; regulating the apoptotic pathways in mitochondria	[25]
	Whey protein	hydrolysate	Neuroprotection	Promote MDA, PC accumulation; enhance SOD, GSH‐Px activities to resist oxidative stress and strengthen endogenous antioxidant defense. Relieve hippocampal neuronal degeneration and apoptosis, elevate AChE activity, inhibit TNF‐α, IL‐1β expression, and upregulate p‐CaMKII and BDNF expressions.	[1]
plants	walnut	QGRPWG, PSRADIY and AYNIPVNIAR	Antioxidation	Effectively eliminate free radicals	[27]
	hazelnut	YYLLVR	Neuroprotection	Regulate the renin‐angiotensin system (RAS), upregulate the ACE2/MAS axis, and promote the expression of BDNF and other substances.	[28]
	Oat Protein	DFVADHPFLF (DF‐10), HGQNFPIL (HL‐8) and RDFPITWPW (RW‐9)	Neuroprotection	Activate the Nrf2‐Keap1/HO‐1 signaling pathway to enhance antioxidant enzyme activity, alleviate oxidative stress, reduce the levels of MDA, AChE and inflammatory factors, and upregulate the expression of Bdnf, Nrf2 and Erg1 genes	[148]
marine	Lamprey buccal gland	VSLNLPYSVVRGEQFVVQA, DIPVPEVPILE, VVQLPPVVLGTFG and VPPPPLVLPPASVK	Anti‐inflammatory, antibacterial and hypotensive	Significantly increase the expression of anti‐inflammatory factors and decrease the expression of inflammatory factors in THP‐1 cells; anti‐Gram‐positive bacteria; ACE inhibitory activity	[30]
	Tuna	Hydrolysate	Anti‐tumor and immune protection	Alleviate 5‐FU‐induced immunosuppression and intestinal mucosal injury	[3]
	shrimp	QMDDQ	Neuroprotection	Improve scopolamine‐induced memory impairment by activating the Notch1 signaling pathway	[31]
others	Insects	Insect bioactive peptides	Antioxidant, ACE inhibition, antidiabetic, anti‐obesity, anti‐inflammatory	Free radical scavenging, enzyme inhibition	[32]

## 3 Challenges to the Bioactivity of FBPs

**FIGURE 2:**
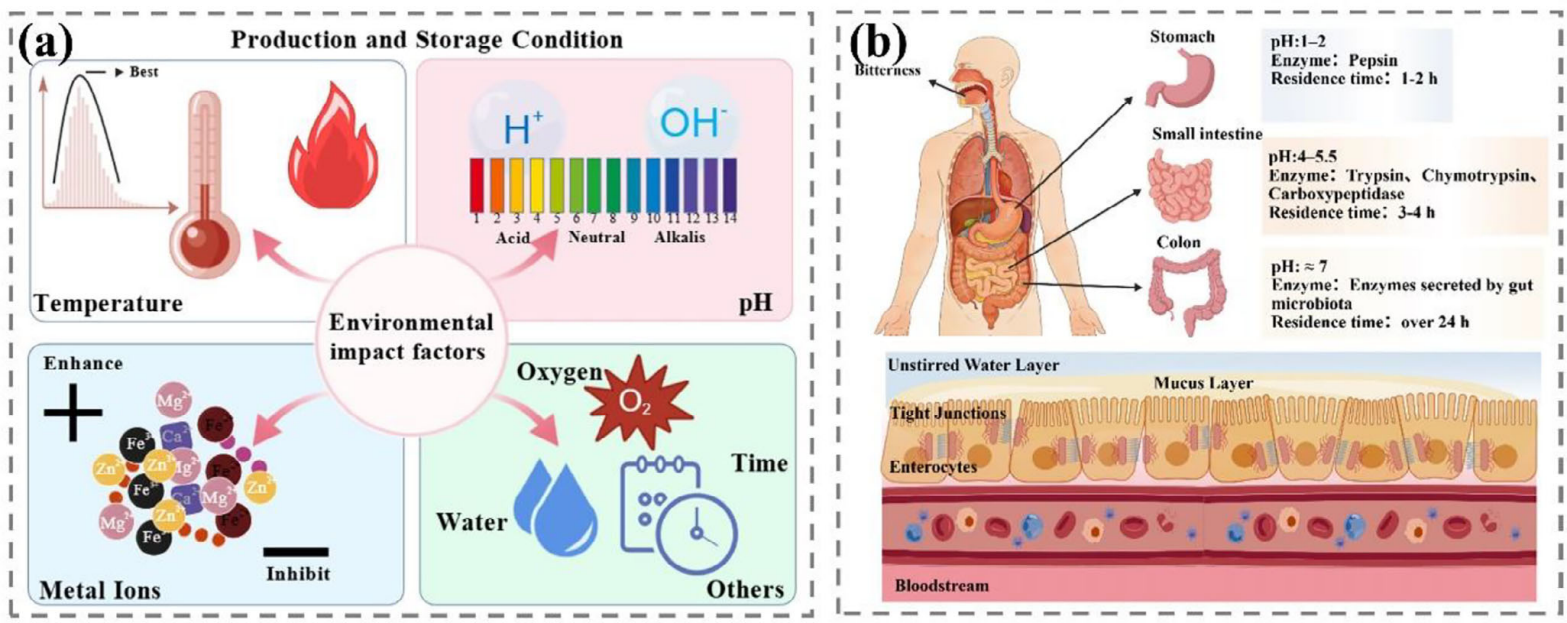
Influencing factors of FBPs. (a) Environmental factors affecting the activity of FBPs. (b) Barriers to oral administration of FBPs. Created with BioGDP.com [147].

The bioactivity of FBPs is susceptible to environmental factors such as temperature, pH, and metal ions during production, processing, storage, and transportation (Figure 2a), as well as bitterness and gastrointestinal environmental conditions during oral administration (Figure 2b). Although oral intake of FBPs offers the advantage of convenience, their bitterness directly impairs palatability and acceptability [33]. More importantly, FBPs need to be absorbed through the gastrointestinal tract in an intact, bioactive form, enter the circulatory system, and accumulate to an effective concentration before exerting their physiological functions [34]. However, this process is subject to degradation by gastric acid and various digestive enzymes (e.g., pepsin, trypsin), which alter peptide structures or cause inactivation, ultimately reducing their bioavailability significantly [34].

### 3.1 Effects of Environmental Factors on FBPs Bioactivity

The bioactivity of FBPs is susceptible to multiple environmental factors during production, processing, storage, and transportation. These factors – temperature, pH, metal ions, oxygen, and humidity – can individually or synergistically alter peptide structure and function. The following subsections detail their respective impacts.

#### 3.1.1 Effects of Temperature on the Stability and Bioactivity of FBPs

Heat treatment exerts an important and complex influence on the FBPs. Generally, high temperatures alter the secondary structures of peptides, leading to aggregation and inactivation [35]. For example, Xi et al. found that long‐term storage at 37°C significantly increased the content of bitter peptides in milk [36]. However, the effect of temperature is not unidirectional; moderate temperature elevation can positively regulate bioactivity under specific conditions. Zeng et al. conducted immunomodulatory experiments on bioactive peptides from Rana spinosa meat and showed that when the enzymatic hydrolysis temperature increased from 35 to 40 °C, the cell proliferation rate peaked at 142.8%. Subsequent further temperature increases led to a gradual decline in the proliferation rate, which dropped to 114.3% at 55 °C [37]. This finding demonstrates that temperature modulates the immune function of macrophages by regulating peptide structures. Similarly, during the enzymatic hydrolysis for preparing functional peptides, temperature generally follows a pattern of initial promotion followed by inhibition. Meng et al. prepared ACE inhibitory peptides from walnut meal protein via alkaline hydrolysis and acid precipitation, and found that the bioactivity first increased and then decreased with rising hydrolysis temperature, with the inhibition rate reaching a peak of 63.65% at 55 °C [38]. It is worth noting that continued temperature elevation or the presence of other components may trigger more complex chemical changes. Ranok et al. studied rice bran hydrolysates in noodles and found that high temperatures might induce peptide aggregation or Maillard reactions with reducing sugars, resulting in increased molecular weight and altered antioxidant properties [39]. These studies indicate that temperature directly affects peptide functions by regulating their structures, and this effect is bidirectional and condition‐dependent, providing a critical basis for optimizing food processing technologies.

#### 3.1.2 Effect of pH on the Stability and Bioactivity of FBPs

pH critically modulates the stability and bioactivity of FBPs. The pH value plays a crucial regulatory role in the stability and biological activity of peptides by altering the protonation state of ionizable side chains in FBPs, profoundly regulating their intramolecular/intermolecular electrostatic interactions, spatial conformation, and exposure to hydrophobic regions [40]. This pH‐driven molecular‐level reconstruction plays a central role in production, activity expression, and storage processes. Research has shown that the pH value of enzyme hydrolysis during peptide production can affect enzyme activity and peptide release. For example, the DPPH free radical scavenging activity of selenium‐enriched Moringa oleifera peptides first increased and then decreased with rising hydrolysis pH, as elevated pH disrupts the active center and spatial structure of enzymes, reducing their catalytic activity [41]. Furthermore, the regulatory effect of pH on peptide bioactivity exhibits significant source dependence. Studies have found that porcine and bovine hydrolysates exhibited the strongest antibacterial activity at pH 2; turkey mince hydrolysates showed optimal anti‐yeast and antibacterial activities at pH 2, whereas bovine hydrolysates achieved the best antibacterial activity at pH 3 [42]. These differences stem from variations in the amino acid sequences of hemoglobin polypeptide chains across species and the structural characteristics of cell walls [43]. Similarly, bioactive peptides from plant and algal sources follow their own distinct pH‐activity profiles. Tomato pomace peptides (TPP) of different molecular weights maintained over 70% stability across all pH conditions, with ACE inhibition rates exceeding 50% under strongly acidic conditions and approaching or exceeding 60% under alkaline conditions. Ghelichi et al. found that antioxidant peptides derived from red algae exhibited better activity under slightly acidic to neutral conditions, while strong alkaline conditions might lead to activity loss [44]. In addition, some peptides exhibit optimal biological activity under neutral pH conditions. Sanchez‐Reinoso et al. investigated the effects of pH values (3, 7, and 10) on the biological activity of hemoglobin peptides in cattle and pigs. pH 7 significantly promoted its antifungal activity [45]. The superior stability of FBPs under neutral pH could be attributed to the avoidance of pH‐induced chemical degradation pathways. Under strongly acidic or alkaline conditions, peptides are susceptible to deamidation of asparagine and glutamine residues, racemization of chiral amino acids, and aggregation driven by increased exposure of hydrophobic regions [46, 47]. These reactions alter peptide conformation, reduce bioactivity, and may generate immunogenic byproducts. Neutral pH minimizes these undesirable modifications, thereby preserving peptide integrity and function. Therefore, neutral conditions are recommended for packaging and storage to maintain optimal bioactivity [7]. In summary, the effect of pH on FBPs bioactivity is source‐dependent and condition‐specific, with complex and critical regulatory roles. Controlling an appropriate pH environment is essential for maintaining peptide stability and function during practical processing and storage.

#### 3.1.3 Effects of Metal Ions on the Stability and Bioactivity of FBPs

Metal ions are among the key factors affecting the stability and function of FBPs. During processing, storage, and transportation, peptides may come into contact with trace metal ions from containers or raw materials. These ions can alter peptide structures and bioactivity by changing the charge environment, inducing complexation, or triggering oxidation reactions.

On the one hand, metal ions can bind to specific sites on peptide chains, causing molecular aggregation; on the other hand, ions such as Fe^2^^+^ may catalyze oxidation reactions, leading to peptide denaturation [48]. In practical systems, the adverse effects of metal ions generally depend on their concentration and type. The ACE inhibitory activity of tomato peptides decreased significantly after the addition of metal ions such as Fe^2^^+^ and Cu^2^^+^, with the inhibitory effect following the order of Fe^2^^+^ > Cu^2^^+^ > Mg^2^^+^ > K^+^. Among these, Fe^2^^+^ reduced the inhibition rate to 52.5%, which was attributed to metal ions disrupting chemical bonds between peptides, reducing solubility, and exposing hydrophobic groups [7]. Similar phenomena have been observed in peptides from different sources. The activity of chicken breast hydrolysates remained stable at low concentrations of Zn^2^^+^, Ca^2^^+^, and Fe^2^^+^, but decreased significantly with increasing ion concentrations, especially in the presence of Fe^2^^+^ and Fe^3^^+^. This proves that metal ions induce aggregation or conformational changes by triggering intermolecular interactions among peptides, thereby affecting bioavailability [48].

Nonetheless, such negative effects arising from structural disruption do not encompass the full scope of metal ion actions. When metal ions match peptide sequences with specific chelating ability, their impact on bioactivity may reverse from inhibition to synergistic enhancement. Studies have shown that when strontium ions (Sr^2^^+^) act in conjunction with deer antler dipeptide YR, the proliferation rate of osteoblasts increased by 12.3%; zinc (Zn^2^^+^) and manganese (Mn^2^^+^) also exhibited certain promoting effects [49]. This positive synergy likely arises from the formation of stable peptide‐metal coordination complexes that facilitate receptor binding or signaling pathway activation, rather than from non‐specific structural disruption.

In summary, the effect of metal ions on peptide bioactivity is highly dependent on ion type, concentration, and peptide sequence context. Most metal ions (e.g., Fe^2^^+^, Cu^2^^+^ at high concentrations) exert negative effects through structural interference and aggregation, while specific combinations (e.g., Sr^2^^+^ with YR) can yield synergistic enhancement. Therefore, during the preparation and storage of peptide products, systematic evaluation of the sources and impacts of metal ions is required to balance their potential risks and functional value.

#### 3.1.4 Other Environmental Factors Affecting FBPs

In addition to temperature, pH, and metal ions, oxygen and humidity in the environment are also key factors affecting FBPs stability. Particularly during the storage of powdered peptide products, hygroscopicity may cause caking, liquefaction, and even oxidative deterioration, thereby reducing nutritional and functional quality. Studies have shown that storage humidity significantly affects the retention of peptide bioactivity. After storage under high humidity conditions (75% RH), the DPPH and ABTS free radical scavenging activities of egg white peptide powder were significantly lower than those stored under low humidity conditions (50% RH). This indicates that air humidity has a significant impact on the antioxidant activity of peptide powders stored at low temperatures, possibly because high humidity environments promote reactions between peptide powders and oxygen, reducing antioxidant activity [50]. This effect does not exist in isolation; in practical storage, humidity often acts synergistically with oxygen and light to accelerate peptide degradation. The decrease in antioxidant activity of porcine heme peptides during storage is also closely related to oxidation and degradation reactions triggered by the combined effects of humidity, oxygen, and light [51]. In summary, oxygen and humidity, as often overlooked environmental factors, synergistically accelerate peptide oxidation and structural changes, thereby reducing bioactivity. Controlling environmental humidity and isolating oxygen are necessary measures to maintain the long‐term stability and functional integrity of peptide products during production, storage, and transportation.

### 3.2 Barriers to Oral Absorption of FBPs

#### 3.2.1 Bitterness

Bitterness is a major sensory barrier restricting the oral application of FBPs, directly affecting product acceptability and consumer willingness [52]. The bitterness intensity is often positively correlated with the content of hydrophobic amino acids, which are precisely the key structural units responsible for bioactivities such as antioxidation and hypotension [53, 54]. Ding et al. prepared tuna blood hydrolysate (TBHN) using neutral protease, which exhibited significant antioxidant and ACE inhibitory activities; its hydrophobic amino acid content reached 60%, resulting in prominent bitterness [54]. Traditional debittering methods such as extensive hydrolysis, membrane separation, or chromatographic separation are often accompanied by bioactivity loss and increased costs [52]. For this reason, research has focused on technological innovations to precisely control bitterness while preserving bioactivity. Huang et al. adopted ultrasonic pretreatment combined with sequential dual‐enzyme hydrolysis, which altered peptide fragment composition and reduced the exposure ratio of bitter amino acids [55]. Meanwhile, innovations in delivery systems have provided physical solutions for masking bitterness. Gao et al. encapsulated bitter peptides in W1/O/W2 double emulsions, reducing particle size, improving physical stability and encapsulation efficiency through aqueous phase gelation, and effectively reducing peptide bitterness intensity [56]. These strategies provide feasible approaches for developing peptide‐based products with both high bioactivity and good palatability, facilitating their application in practical food and health fields.

#### 3.2.2 PH Gradient and Enzymatic Hydrolysis Effects

The pH values and various digestive enzymes in the gastrointestinal tract constitute major biochemical barriers for orally administered bioactive peptides. Generally, the stomach is the most acidic region in the human body, with a pH range of 1–3. In the duodenum, the pH increases to 4–5.5 due to the neutralizing effects of carbonate and bile acids, while the pH in the rectum and colon is maintained at approximately 7. The bioactivity of peptides depends on their specific amino acid sequences and spatial conformations, which can be altered under different pH conditions. In the gastric acid environment, some peptide chains may undergo degradation or denaturation, as the acidic conditions disrupt hydrogen bonds and hydrophobic interactions within peptide molecules, potentially leading to structural instability [57]. In addition, various enzymes in the gastrointestinal tract pose significant obstacles to bioactive peptides, thereby reducing their in vivo bioavailability [58]. These mainly include enzymes in saliva, digestive enzymes in gastric juice, and pancreatic enzymes in the small intestine, each exerting specific functions under different conditions. When peptides enter the gastric acid environment, their structures denature, and pepsin is subsequently activated. Pepsin can cleave peptide bonds in proteins, breaking them down into smaller peptide fragments with shorter chain lengths [59]. These smaller peptide fragments, along with partially digested proteins, are transported to the small intestine together with other gastric contents. The pancreas secretes proteolytic enzymes including trypsin, chymotrypsin, and carboxypeptidase into the small intestine, further breaking down peptides into shorter fragments and amino acids [60]. The final stage of protein digestion occurs at the brush border of the small intestine, where enzymes such as aminopeptidase and dipeptidase continue to decompose peptides into individual amino acids [61]. The resulting amino acids and small‐molecule peptides are absorbed through the epithelial cells of the small intestinal wall into the bloodstream and then transported to various tissues throughout the body for growth and repair processes [62].

However, unprotected peptides often lose substantial bioactivity before reaching the absorptive sites due to the aforementioned pH fluctuations and enzymatic hydrolysis. Consequently, the pH gradient along the gastrointestinal tract and the sequential action of digestive enzymes provide the key physiological rationale for designing pH‐responsive release carriers and enzyme inhibitors, which aim to protect peptides from degradation and achieve site‐specific delivery.

#### 3.2.3 Intestinal Mucus Layer and Epithelial Barrier

After successfully passing through the unstable pH environment and enzymatic hydrolysis by digestive enzymes in the gastrointestinal tract, the physical‐chemical composite structure of the intestinal barrier forms a second core barrier. To be absorbed through cell membranes, bioactive peptides must first cross the unstirred water layer after entering the small intestine. The unstirred water layer is an extracellular protective structure covering the surface of intestinal microvilli, and its thickness increases with higher intestinal viscosity and weaker mixing effects. Increased thickness limits the mass transfer rate between the intestinal lumen and absorption sites, hindering peptide diffusion [63]. Intestinal mucus is a gel‐like structure formed by mucins on the surface of intestinal epithelial cells. Under normal physiological conditions, the pore size of the mucus layer typically ranges from approximately 20 to 200 nm, although it can expand to up to 1800 nm under certain pathological or inflammatory states [64]. The surface of the mucin network contains densely packed hydrophilic and hydrophobic regions, which generate hydrophobic interactions with peptides, impeding peptide diffusion within the mucus and forming a hydrophobic network barrier [65]. In addition, mucus forms various low‐affinity interactions with peptides on its surface, which also restrict peptide diffusion, creating a physical‐chemical interaction barrier [66]. Intestinal mucus can be divided into a loosely adherent mucus layer and a firmly adherent mucus layer. The loosely adherent layer continuously secretes and sheds mucins, resulting in complete mucus renewal every 4–5 h. Although this mechanism for rapidly clearing adherent bacteria or pathogenic substances is beneficial for intestinal health, it shortens the retention time of peptides in the intestinal tract, thereby forming a physical filtration barrier and significantly reducing peptide uptake. The firmly adherent layer hinders macromolecule penetration through the steric hindrance and charge effects of the mucin network [10]. After overcoming multiple obstacles including adhesion and pre‐degradation by the intestinal mucus layer, peptides must cross the small intestinal epithelial barrier to enter the circulatory system. The transmembrane transport mechanisms include carrier‐mediated transport, paracellular transport, and endocytosis [62]. The cell membrane of the intestinal epithelial layer has a lipid bilayer structure, which prevents foreign substances from entering the bloodstream. Hydrophilic peptides are mainly transported across the intestinal epithelium via the paracellular pathway, which is restricted by tight junctions (TJs) – an important component of the intestinal epithelial layer. Only molecules with a hydrodynamic radius smaller than 1 nm can penetrate tight junctions, which directly limits the transcellular transport of peptides [57].

In summary, before exerting physiological effects on target tissues, FBPs must overcome not only bitterness but also various enzymatic systems, pH environments, and absorption barriers in the digestive tract to enter the circulatory system. In the gastrointestinal tract, bioactive peptides suffer reduced bioavailability, are prone to degradation, and are difficult to absorb by the human body [62]. Therefore, it is imperative to develop technical methods that can effectively mask peptide bitterness, protect their bioactivity, enable complete absorption, and improve bioavailability.

## 4 Delivery Systems

**FIGURE 3:**
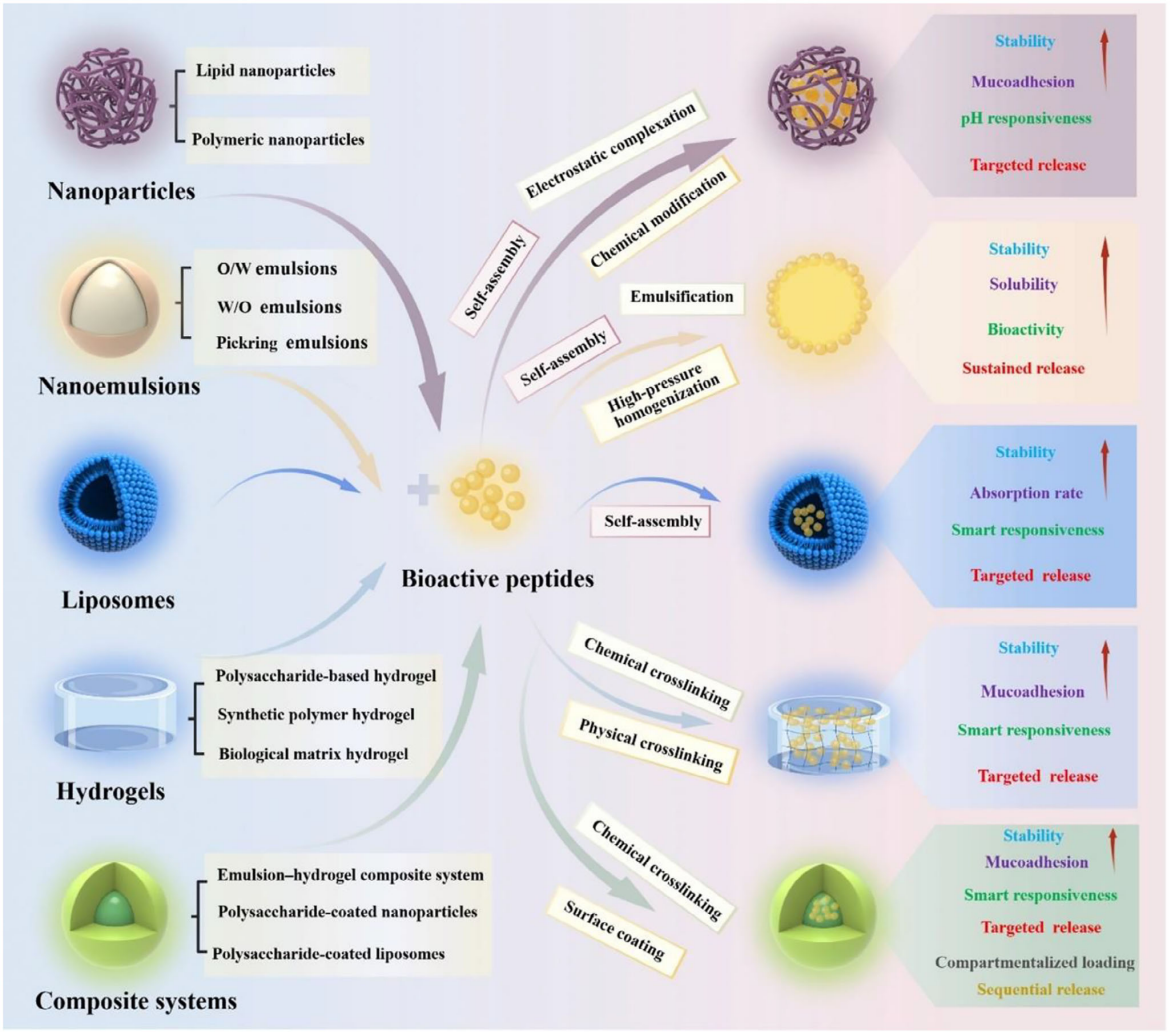
Types of main delivery systems, preparation methods, and core performance advantages of each system for bioactive peptides. Created with BioGDP.com [147].

Given the limitations of oral peptides, such as bitterness and low bioavailability caused by multiple gastrointestinal barriers, the development of effective delivery carriers has become the key to enhancing their functional value. Delivery systems represented by nanoparticles, nanoemulsions, liposomes, hydrogels, and composite systems can effectively mask bitterness, resist enzymatic hydrolysis, and promote intestinal absorption through physical encapsulation and structural modification, thereby significantly improving the bioavailability and physiological activity of peptides (Figure 3) [67]. Table 2 summarizes the differences among various delivery systems in encapsulating different FBPs. It is important to note that different FBPs vary widely in their effective doses, molecular properties, and susceptibility to gastrointestinal degradation. Therefore, the selection of a delivery system (e.g., nanoparticles, liposomes, hydrogels) should be rationally matched with the specific absorption requirements of the peptide.

**TABLE 2:** Comparison of different oral delivery systems for food‐derived bioactive peptides (FBPs).

System	Material	Peptide	EE/EL	Results	References
Nanoparticles	Lipid	Oat Globulin Peptide	69.8 – 75.6%	Enhanced storage stability, ensuring the stability of peptides in SGF.	[68]
	PLGA, Lipid, PEG_2000_‐Chol	Plant peptide KY5	89.88 ± 1.23% / 1.54 ± 0.25%	Good stability with controlled‐release behavior in the gastrointestinal tract; it enhances in‐situ intestinal absorption and improves oral absorption as well as antihypertensive efficacy.	[69]
	Chitosan‐Flaxseed Gum	Bighead carp peptide (BCP)	60.3 ± 4.1% / 23.4 ± 2.8	Enhanced thermal stability; controlled release of BCP in a dose‐dependent manner following the first‐order kinetic model	[71]
	Thiolated chitosan	Marine crab myogenic peptide (ESPVL)	88.19 ± 2.34% / 72.41 ± 1.99%	The peptide achieves sustained release for up to 30 h, with improved stability and potent anti‐inflammatory activity.	[72]
	Trimethylated chitosan‐sodium alginate	Peanut peptide (PP)	91.14 ± 4.82% / 4.80 ± 0.99%	The antioxidant activity is significantly enhanced; it inhibits the release of PP in simulated gastric medium and promotes the release of PP in simulated intestinal medium.	[73]
	Modified starch—lecithin	β‐lactoglobulin peptide (β‐LG)	50%	Good mucus penetration capacity (59.25%); colon‐targeting ability (49.18% released in simulated colonic fluid); significant promotion of glucagon‐like peptide‐1 (GLP‐1) secretion (an increase of 119.23%)	[76]
Nanoemulsions	Oil‐in‐water (O/W) emulsion	Casein Peptide	Not reported	Improve the physical stability of the lotion and achieve dual antioxidant protection effects	[81]
	Zn^2+^‐induced self‐assembled amphiphilic emulsion	Oat peptide (CEO)	95%	Enhanced stability; strong controlled‐release capacity to effectively inhibit the growth of spoilage microorganisms	[82]
	Proanthocyanidins (PC) self‐assembled O/W emulsion	Egg white peptide (EWP)	Not reported	Enhance the emulsifying performance and stability of peptides	[83]
	Pickering emulsion	Soybean peptide	Not reported	Enhance the interfacial adsorption capacity and antioxidant activity of the system	[84]
	Xanthan gum	Egg white gel‐derived peptides	Not reported	Increased stability in alkaline environment; enhanced antioxidant capacity	[85]
Liposomes	Cholesterol and lecithin	Corn peptide (MP)	86.16 ± 2.29% / 21.52 ± 1.74%	It can significantly improve the in vitro digestibility and cellular absorption utilization rate of peptides, and also enhance the body's antioxidant capacity and reduce the cell apoptosis rate by regulating the expression of antioxidant‐related genes.	[87]
	Phospholipids and cholesterol	Soy protein peptide	60.5%	Achieves gastrointestinal sustained release and peptide stability	[89]
	Lecithin and cholesterol	Casein peptide (CP)	87.29 ± 0.82%	Prevent degradation by gastrointestinal enzymes and achieve controlled release in the intestines	[90]
	Lecithin and cholesterol	Defatted flaxseed polypeptide	84–87%	The peptide encapsulation efficiency decreases by approximately 20–30% in the simulated gastric environment and around 70–80% in the simulated intestinal environment	[91]
	Soy Lecithin	Salmon skeleton protein hydrolysate (SFPH)	89.63%	Effective masking of bitter taste	[92]
	Hydroxyapatite (HAP), Soy Lecithin, Cholesterol	Walnut peptide (WPs)	76.7 ± 0.03%	HAP effectively inhibits the hydrolysis of phospholipids and reduces the damage to lipid bilayers, endowing pro‐liposomes with sustained‐release properties.	[93]
Hydrogel	Chitosan‐sodium alginate‐sodium tripolyphosphate	*Ganoderma lucidum* peptide (GLP)	81.73%	The amounts of peptides released from hydrogels in simulated gastric fluid are less than 30%, and both digestive stability and thermal stability are improved.	[102]
	Carboxymethyl cellulose (CMC)—polyvinyl alcohol (PVA)	Soy peptide (SPE)	Not reported	Only a small amount of SPE is released in SGF, while SPE is completely released in SIF. The scavenging rates of the released SPE against DPPH and ABTS free radicals are 41.68% and 31.43%, respectively.	[103]
	Chitosan‐alginate	Rice bran active peptides (RBAP)	Not reported	Sustained release of more than 70% peptides under intestinal conditions; the free radical scavenging capacity is significantly increased by 3 to 7 times, and it also protects human umbilical vein endothelial cells from H2O2‐induced oxidative stress after digestion	[18]
	Peptide self‐assembly	Ovalbumin‐derived peptides VLVNAIVFKGL	Not reported	Hydrogelation of fibrous networks with high fiber stability to reduce ROS production	[105]
	NIPAAm‐*co*‐(N‐acryloxysuccinimide)*‐co‐* (polylactide/‐hydroxy methacrylate)*‐co‐* (oligo (ethylene glycol))) (PNPHO)	Ox horn peptide	Not reported	Promote the formation of new bone tissue and establish the vascular network essential for sustained tissue regeneration	[106]
	Costal chondrocyte extracellular matrix (ECM)	Melittin	Not reported	Reduce the inherent toxicity of melittin and prolong its release behavior simultaneously	[107]
Composite systems	Whey Protein Isolate (WPI)/Soy Protein Isolate (SPI) composite particles stabilize Pickering Emulsion (PE), and sodium alginate (SA) is added to form Pickering Emulsion Gel (PEG)	Milk source peptide FDRPFL	72.8%	Improve the bioaccessibility of peptide FDRPFL, and it mainly follows the release mechanism of non‐Fickian superstate II	[108]
	Rice bran wax and W/O emulsion constructed water‐in‐oil emulsion filled hydrogel beads	Collagen peptide	85.18±0.46%	Effectively enhance the degree of delayed release of free fatty acids and peptides	[109]
	Sodium tripolyphosphate (TPP) cross‐linked chitosan‐coated alginate nanoparticles (PL‐C‐AL‐NP)	Stonefish Peptide ALGPQFY	74.48%/16.67%	Improve the stability, bioavailability, and antihypertensive efficacy of ALGPQFY	[110]
	Sodium alginate‐coated liposomes	Collagen peptide	70.99%	Gastrointestinal stability increased by 50%, transcellular permeability rose by 18%, and in vitro release rate decreased by 34%	[19]
	Carboxymethyl chitosan (CMCS) as the shell and γ‐cyclodextrin (γ‐CD) as the core to form composite nanoparticles	Egg white peptide EWDP	15.3–26.8%/7.0–12.2%	Enhance antioxidant activity, bioaccessibility and gastrointestinal stability; the absorption capacity of Caco‐2 cells is significantly improved in the presence of EWDP (an increase of over 20%)	[111]

### 4.1 Nanoparticles

Nanoparticles function as efficient carriers for the oral delivery of peptides primarily by protecting bioactive peptides from degradation in the gastrointestinal environment through physical encapsulation and structural stabilization, while enhancing their transmembrane absorption and targeted delivery efficiency [4]. Nanoparticles are colloidal systems with sizes ranging from 10 to 1000 nm, usually constructed from materials such as lipids, polysaccharides, starch, or synthetic polymers via self‐assembly, emulsification, polyelectrolyte complexation, and other methods. They possess characteristics including small size, large specific surface area, and strong surface modifiability.

**FIGURE 4:**
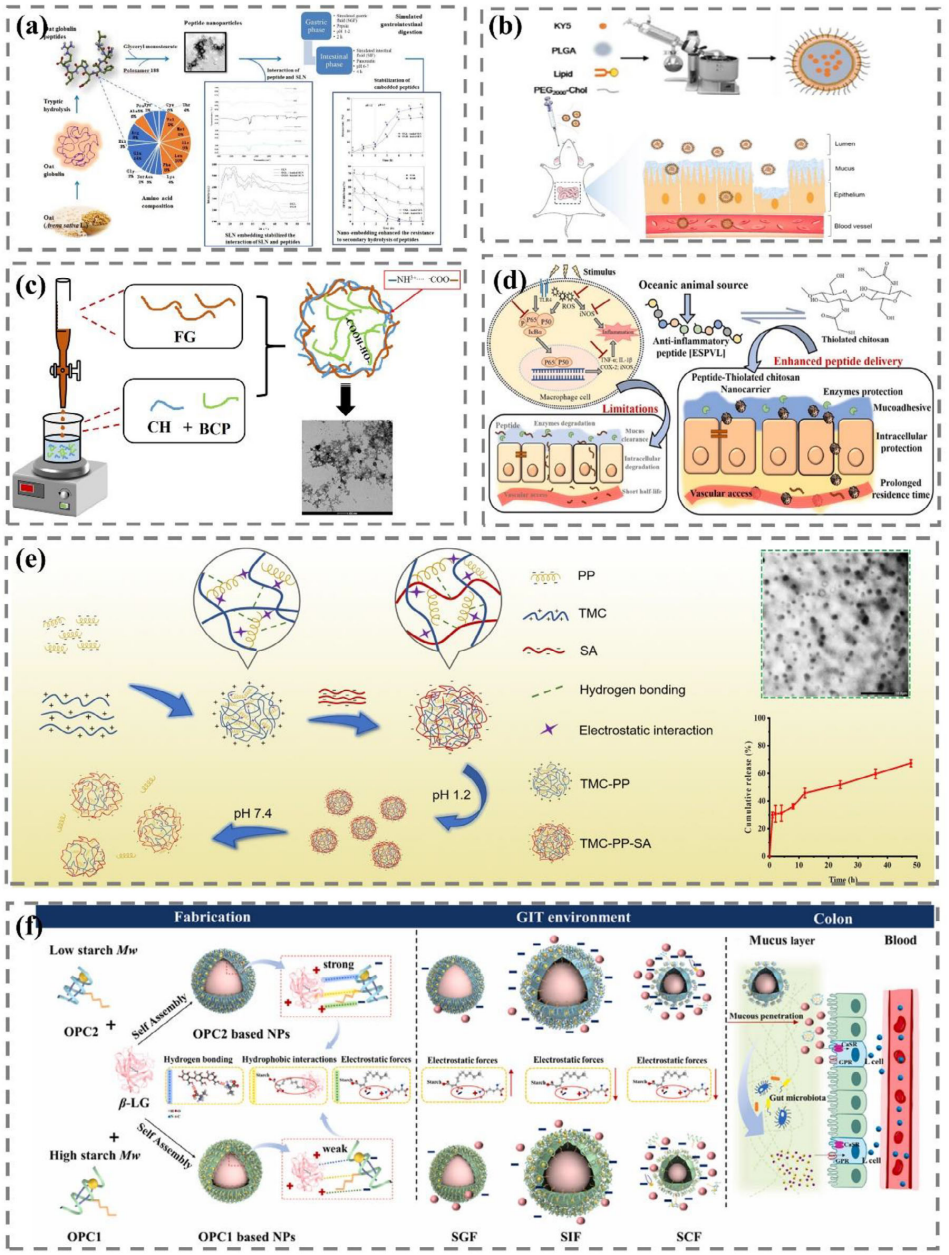
Nanoparticle delivery of FBPs. (a) Lipid nanoparticles embedding oat globulin peptides. Reproduced with permission [68]. Copyright 2020, Elsevier Publishing Group. (b) Lipid‐polymer nanoparticles (KY5‐CSs) delivering plant peptide KY5. Reproduced with permission [69]. Copyright 2023, Elsevier Publishing Group. (c) Chitosan‐flaxseed gum polyelectrolyte nanoparticles (CS‐FG‐NP) encapsulating bighead carp peptides (BCP). Reproduced with permission [71]. Copyright 2023, Elsevier Publishing Group. (d) Thiolated chitosan nanoparticles (Pep‐TCS NP) loading marine crab myogenic peptide (ESPVL). Reproduced with permission [72]. Copyright 2023, Elsevier Publishing Group. (e) N‐[(2‐hydroxy‐3‐trimethylammonium) propyl] chitosan‐sodium alginate nanoparticles encapsulating peanut peptides (PP). Reproduced with permission [73]. Copyright 2022, Elsevier Publishing Group. (f) Modified starch‐lecithin nanoparticles (OPC) encapsulating β‐lactoglobulin peptides (β‐LG). Reproduced with permission [76]. Copyright 2025, Elsevier Publishing Group.

Studies have shown that various peptides exhibit significantly enhanced functions after being encapsulated in nanoparticles. Lipid nanoparticles have become commonly used carriers due to their excellent biocompatibility and encapsulation capacity. Su et al. prepared lipid nanoparticles using lipid materials such as triglycerides to encapsulate oat globulin peptides (Figure 4a). Oat peptides encapsulated in nanoparticles maintained high bioactivity levels in a simulated gastric fluid environment, demonstrating that these nanoparticles can effectively ensure the stability and bioactivity of peptides in simulated gastric juice [68]. This study reveals the physical encapsulation protection mechanism of lipid nanoparticles. However, the differences between simulated gastric fluid and dynamic real gastrointestinal conditions, as well as the impact of lipid digestion on peptide release behavior, require further investigation.

Furthermore, Yuan et al. developed core‐shell lipid nanoparticles (KY5‐CSs) for the oral delivery of KY5, a highly potent antihypertensive peptide derived from plants (Figure 4b). This system not only exhibited uniform distribution and high stability but also enhanced mucus penetration and cellular uptake, significantly improving the oral absorption efficiency and functional efficacy of the antihypertensive peptide in a rat model [69]. This study demonstrates the dual value of lipid nanoparticles in enhancing bioavailability. However, the interfacial stability of the core‐shell structure and its structural rearrangement mechanisms during trans‐mucosal transport require systematic elucidation.

In comparison, polysaccharide‐based polymeric nanoparticles stand out prominently. Chitosan is frequently used to construct polymeric carriers due to its outstanding biocompatibility, mucoadhesiveness, and ability to open tight junctions [70]. Zheng et al. utilized molecular self‐assembly of a chitosan‐flaxseed gum polymer (CS/FG NP) to form uniform spherical structures, which served as delivery carriers for bighead carp peptides (BCP), resulting in the formation of CS/FG‐BCP nanoparticles (Figure 4c). This system enhanced stability through hydrogen bonding and electrostatic interactions, enabling controlled release and thermal stability of BCP [71]. This strategy demonstrates the synergistic stabilization effect of polysaccharide complexes. However, whether the cationic nature of chitosan may induce non‐specific trapping in the mucus layer and its potential impact on gut microbial ecology require attention in subsequent studies.

Furthermore, the biological and chemical properties of chitosan can be enhanced through chemical modification. Balde et al. prepared thiolated chitosan nanoparticles (Pep‐TCSNP) that exhibited improved bioadhesion and chemical stability via thiolation modification. After loading ESPVL, an anti‐inflammatory peptide derived from marine crab muscle, the nanoparticles achieved sustained and stable release for 30 h (Figure 4d), significantly enhancing the stability and thermal stability of the peptide at target sites and demonstrating excellent inflammatory regulation activity at the cellular level [72]. The innovation of this study lies in utilizing disulfide exchange with mucus glycoproteins to enhance mucosal retention. However, the balance between the degree of thiolation and nanoparticle toxicity, as well as their stability in the reducing intestinal environment, requires systematic optimization.

Beyond chitosan, other polysaccharides such as sodium alginate have also been successfully used to construct intelligent delivery systems. Han et al. innovatively constructed a pH‐responsive release carrier formed by electrostatic self‐assembly of two polysaccharides, N‐trimethyl chitosan and sodium alginate, and applied it for encapsulating peanut peptides (PP) (Figure 4e). This system inhibited the rapid release of peanut peptides in the acidic gastric environment and promoted efficient release in the neutral intestinal environment, resulting in significantly enhanced antioxidant activity, an approximate 41.76% increase in intestinal absorption rate, and no cytotoxicity, laying the foundation for safe applications [73]. This design demonstrates the unique advantages of polysaccharide composites in environmentally responsive delivery. However, the regulatory effects of charge density and molecular weight of the two polysaccharides on assembly structure and pH response thresholds require in‐depth investigation.

Starch is widely used in nano‐delivery systems due to its low cost, high biocompatibility, and ease of modification [74]. Modification of natural starch can improve its inherent properties, thereby expanding its application scope in the food industry and other fields [75]. Wang et al. prepared modified starch‐lecithin complex (OPC) nanoparticles via intermolecular interactions and self‐assembly strategies for encapsulating β‐lactoglobulin peptides (β‐LG) (Figure 4f). Nanoparticles constructed from low‐molecular‐weight starch exhibited good upper gastrointestinal tract protection and colon‐targeted release capability (49.18%), effectively promoting intestinal hormone secretion (119.23%) and enhancing mucus penetration efficiency (59.25%) [76]. This study provides a feasible approach for colon‐targeted peptide delivery. However, the potential impact of starch modification processes on peptide structural integrity, as well as the mechanisms of carrier degradation and utilization by colonic microbiota, require systematic evaluation.

In summary, current research has systematically explored the applications of polysaccharides, lipids, and composite materials in peptide delivery, covering aspects such as material modification, self‐assembly processes, release kinetics regulation, and biological effect enhancement. It has been confirmed that technologies including lipid diffusion, polyelectrolyte complexation, and intermolecular self‐assembly can achieve efficient peptide encapsulation, gastric protection, intestinal targeted release, and functional enhancement. However, most existing studies focus on the delivery optimization of single peptides; in‐depth investigations are still needed on the synergistic mechanisms of polypeptide co‐delivery systems, long‐term storage stability, and scalability for industrial production. In addition, the interaction mechanisms between nanoparticles and gut microbiota, differences in release behavior under different physiological conditions, and the balance between biodegradability and environmental safety remain important directions for future research. These explorations will provide theoretical support for developing efficient, safe, and intelligent peptide delivery carriers, promoting their wide application in functional foods, nutritional interventions, and health regulation.

### 4.2 Nanoemulsions (NEs)

Nanoemulsions (NEs) are high‐efficiency oral delivery systems that form nanoscale droplets (10‐1000 nm) to effectively encapsulate bioactive peptides, thereby enhancing their solubility, stabilizing their structures, and promoting intestinal absorption to improve bioavailability [77, 78]. This system is characterized by a large specific surface area and uniform dispersion, which not only improves the dissolution and release behavior of bioactive substances but also effectively resists adverse factors during processing, storage, and gastrointestinal transit (such as pH changes and enzymatic hydrolysis). Meanwhile, it helps mask off‐flavors and optimize sensory quality [79, 80].

**FIGURE 5:**
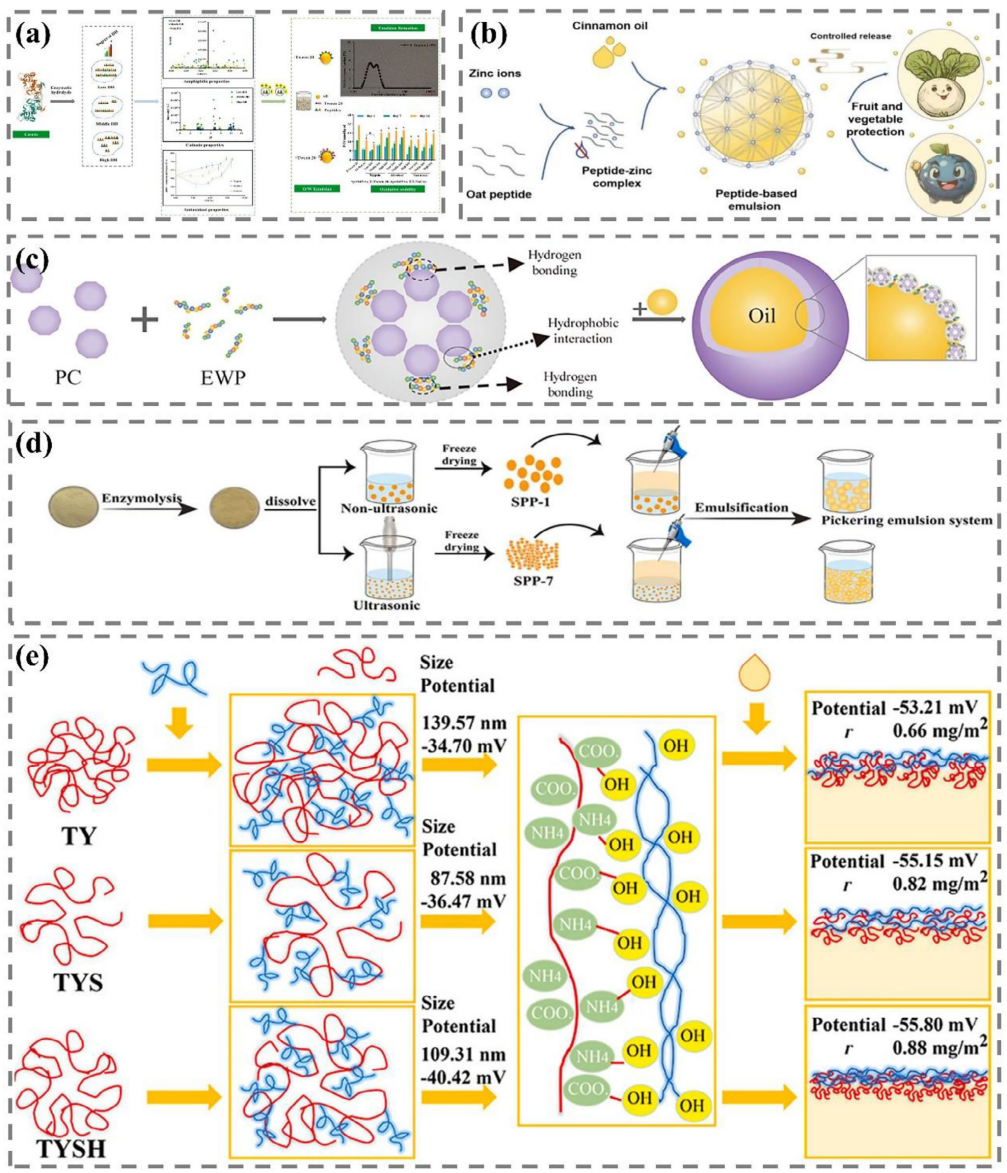
Nanoemulsion delivery of FBPs. (a) Casein peptides enhance the antioxidant properties and stability of emulsions. Reproduced with permission [81]. Copyright 2022, Elsevier Publishing Group. (b) Oat peptides (CEO) form amphiphilic emulsions through zinc ion‐induced self‐assembly. Reproduced with permission [82]. Copyright 2025, Elsevier Publishing Group. (c) Egg white peptides (EWP) and proanthocyanidins (PC) assemble to form aggregates, which are applied in O/W emulsions. Reproduced with permission [83]. Copyright 2023, Elsevier Publishing Group. (d) Pickering emulsion encapsulates soy peptides. Reproduced with permission [84]. Copyright 2025, Elsevier Publishing Group. (e) Xanthan gum forms an emulsion with egg white gel‐derived peptides. Reproduced with permission [85]. Copyright 2022, Elsevier Publishing Group.

Studies have shown that bioactive peptide fragments from specific sources can directly stabilize emulsion interfaces through their structural characteristics. Yu et al. found that specific antioxidant peptide fragments derived from casein could form stable emulsions at an appropriate degree of hydrolysis (Figure 5a). These peptides not only enhanced the physical stability of the emulsions but also provided dual antioxidant protection through their specific amino acid sequences [81]. This finding reveals the dual functionality of peptides as both active ingredients and interface stabilizers. However, the structure‐activity relationship between the degree of hydrolysis and peptide interfacial adsorption capacity, as well as their adaptability to different oil phase systems, requires systematic investigation.

Furthermore, more sophisticated delivery systems can be constructed via metal ion‐induced self‐assembly. Yang et al. discovered that oat peptides (CEO) could undergo zinc ion‐induced self‐assembly to form amphiphilic emulsions with a network structure (Figure 5b), achieving a peptide encapsulation efficiency of up to 95% and controlled release [82]. The innovation of this strategy lies in utilizing metal coordination to drive peptide conformational transitions, constructing a “carrier‐activity” integrated delivery system. This synergy aligns with the positive aspect of the dual effects of metal ions on peptide activity discussed earlier, further demonstrating that the net outcome – whether detrimental or beneficial – depends critically on metal ion type, concentration, peptide sequence, and assembly conditions. However, the influence of zinc ion coordination sites and binding constants on assembly stability, as well as the potential regulatory effects of metal ion release on the intestinal environment, requires in‐depth analysis.

However, many small‐molecule bioactive peptides have limited emulsifying capacity and require molecular interactions or aggregation to improve their interfacial properties. Egg white peptides (EWP, MW < 1 kDa) possess various biological functions, but their application is limited by poor emulsifying performance. Wen et al. bound individual EWP molecules to proanthocyanidins (PC) through hydrogen bonding and hydrophobic interactions, and the formed PC‐EWP complexes subsequently bound more EWP molecules via hydrogen bonding to form PC‐EWP‐EWP aggregates (Figure 5c). When these aggregates were synergistically applied in O/W emulsions, they overcame the bottleneck of poor emulsifying properties of natural peptides and significantly improved emulsion stability by precisely regulating interfacial properties [83]. This strategy demonstrates the synergistic interfacial stabilization effect of polyphenol‐peptide complexes. However, the influence of PC‐EWP binding ratio and sequence on aggregate structure and interfacial behavior, as well as their dissociation kinetics in the gastrointestinal environment, requires further investigation.

On this basis, combining physical or chemical modification technologies can further enhance the function and robustness of peptide‐stabilized emulsions. Han et al. sonicated insoluble soybean peptide aggregates (SPP) and added them to emulsions to form SPP‐stabilized Pickering emulsions (Figure 5d), which exhibited excellent storage stability. The improved performance was attributed to the incorporation of peptides, which enhanced interfacial adsorption capacity and antioxidant activity [84]. Ultrasonic treatment disrupted the original aggregation state of SPP through cavitation effects, exposing more hydrophobic regions and thereby improving interfacial activity. However, the structure‐activity relationship between ultrasonic parameters and SPP structural rearrangement, as well as its universality for peptides of different molecular weights, requires systematic optimization.

To address more severe environmental challenges, the application of composite and homogenization technologies can achieve breakthroughs. Ai et al. formed a stable carrier system by combining xanthan gum with peptides derived from egg white gel, and prepared nanoemulsions using high‐pressure homogenization technology (Figure 5e), breaking through the stability bottleneck of active peptide delivery under alkaline conditions (pH 11.0). This was achieved by altering peptide spatial structures to induce the formation of cross‐linked entangled aggregates, which significantly improved emulsion ionic stability, temperature tolerance, and long‐term storage stability. Moreover, it inhibited lipid oxidation and endowed the emulsions with antioxidant properties [85]. This study provides a feasible solution for peptide delivery under extreme pH conditions. However, the interaction mechanism between xanthan gum and peptides, as well as the potential impact of high‐pressure homogenization on peptide secondary structure, requires in‐depth elucidation.

In summary, nanoemulsions not only provide an effective protection and delivery platform for bioactive peptides but also can synergize with the functional characteristics of peptides during their construction to achieve multiple benefits of “delivery‐stabilization‐function”. However, further exploration is required into the peptide delivery potential of more emulsion systems, the synergistic effects in complex systems, and stability performance under different environmental conditions, as well as expanding their applications in functional foods, pharmaceuticals, and cosmetics to realize the efficient utilization and widespread application of bioactive peptides.

### 4.3 Liposomes

As peptide delivery carriers, liposomes possess the core advantage of simulating the phospholipid bilayer structure of cell membranes. They can simultaneously encapsulate hydrophilic and hydrophobic active ingredients, effectively isolate them from the gastrointestinal digestive environment, protect peptide integrity, and enhance absorption efficiency and bioavailability [4]. Liposomes are mainly composed of biocompatible lipids such as phospholipids and cholesterol, and self‐assemble into nano‐ or micro‐scale vesicles via thin‐film hydration, sonication, and other methods. Reducing their particle size to the nanoscale not only improves encapsulation efficiency but also enhances system stability and functionality, enables precise regulation of peptide release rates, and ultimately significantly improves peptide bioavailability and optimizes liposome pharmacokinetic properties [86].

**FIGURE 6:**
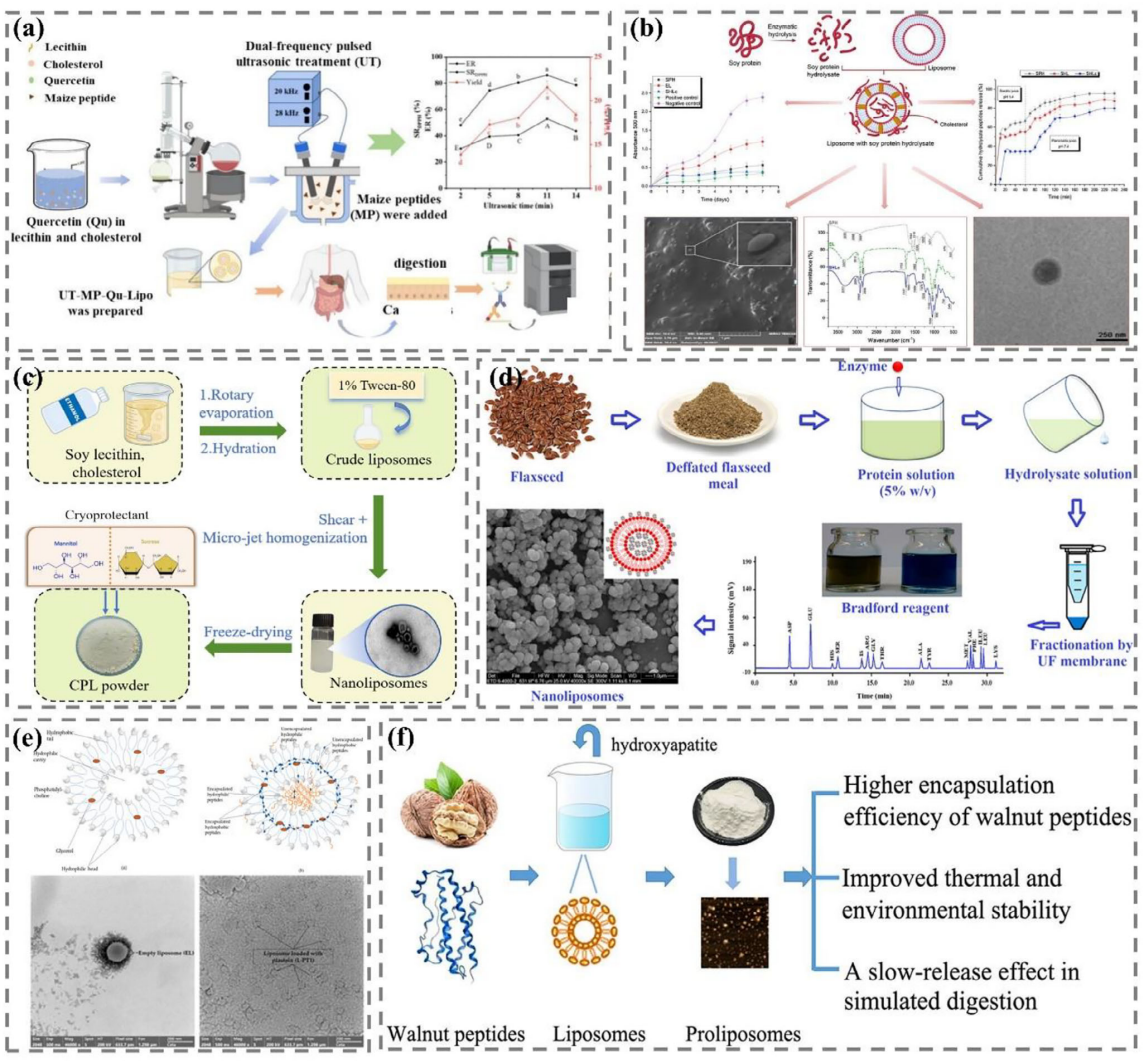
Liposome delivery of FBPs. (a) Liposomes (MP‐Qu‐Lipo) co‐encapsulate maize peptides (MP) and quercetin (Qu). Reproduced with permission [87]. Copyright 2025, Elsevier Publishing Group. (b) Liposomes encapsulate soy protein peptides. Reproduced with permission [89]. Copyright 2022, Elsevier Publishing Group. (c) Liposomes encapsulate casein peptides. Reproduced with permission [90]. Copyright 2025, Elsevier Publishing Group. (d) Liposomes encapsulate defatted flaxseed polypeptides. Reproduced with permission [91]. Copyright 2020, American Chemical Society Publishing Group. (e) Liposomes encapsulate salmon frame protein hydrolysate (SFPH) and plasticized protein (SFPP). Reproduced with permission [92]. Copyright 2023, MDPI Publishing Group. (f) Pre‐liposomes constructed with hydroxyapatite (HAP) encapsulate walnut peptides (WPs). Reproduced with permission [93]. Copyright 2022, Elsevier Publishing Group.

Researchers have adopted various strategies to optimize liposomes for adapting to the delivery requirements of different peptides. Among these, optimizing physical parameters through process innovation represents a fundamental approach to achieving efficient encapsulation and functional enhancement. Qu et al. synthesized corn peptide‐quercetin nanoliposomes (MP‐Qu‐Lipo) using multi‐frequency pulsed ultrasound technology (UT), realizing the co‐encapsulation of corn peptides (MP) and quercetin (Qu) (Figure 6a). Parameter optimization increased encapsulation efficiency by 43.15%, reduced particle size by 87.50%, and improved DPPH free radical scavenging activity by 24.38%, activating the Nrf2/HO‐1 antioxidant pathway to enhance cellular protective effects [87]. Notably, moderate ultrasound treatment can enhance peptide release efficiency and bioaccessibility without causing irreversible structural damage [88], whereas excessive ultrasound may directly disrupt covalent bonds in the peptide backbone, leading to cleavage of the primary structure and the loss or rearrangement of ordered secondary structures such as α‐helices and β‐sheets, thereby reducing peptide bioactivity. However, most existing studies focus primarily on functional indicators such as encapsulation efficiency and antioxidant activity, without systematically evaluating changes in the primary and secondary structures of peptides before and after ultrasound treatment. Future research should prioritize establishing quantitative relationships between ultrasound parameters and peptide structural integrity, thereby providing a theoretical basis for the rational design of ultrasound‐assisted liposome preparation processes.

Furthermore, optimizing the composition and structure of liposomes themselves can strengthen their stability in biological environments. Pavlović et al. constructed a phospholipid‐cholesterol bilayer system and combined it with sonication to encapsulate soybean protein peptides (Figure 6b), achieving an encapsulation efficiency of 60.5% and forming spherical structures with uniform particle size and stable zeta potential. Hydrophobic interactions enhanced stability and prolonged gastrointestinal release time [89]. This study confirms the core role of cholesterol in regulating membrane fluidity and reducing hydrolase permeability. However, the balance between cholesterol content and liposome rigidity, as well as its encapsulation universality for peptides with different hydrophobicities, requires further investigation.

In addition to physical stability, liposome design must also consider interactions with biological systems. Gong et al. optimized the preparation of casein peptide liposomes via thin‐film hydration using response surface methodology (Figure 6c), achieving an encapsulation efficiency of 87.29% and an average particle size of 86.13 nm. These liposomes can not only resist digestive enzyme degradation through the lipid bilayer barrier but also promote directional interactions with gut microbiota to achieve nutrient absorption and microecological regulation [90]. The innovation of this study lies in expanding delivery system design from “passive protection” to “active interaction.” However, the relationship between liposome surface properties and specific microbiota recognition mechanisms, as well as their long‐term impact on microbial ecological networks, requires systematic elucidation.

Regarding structure‐function relationships, the precise localization of peptide molecules within liposomes is crucial for their stability and release behavior. Sarabandi et al. found that after loading defatted flaxseed polypeptides into nanoliposomes, peptide molecules localized in the polar regions of the bilayer, and the spherical structure of liposomes ensured gastrointestinal stability (Figure 6d). This study confirmed, through spectroscopic techniques such as FTIR, the hydrogen bonding interactions between peptides and phospholipid head groups, providing experimental evidence for understanding peptide distribution patterns within liposomes [91]. However, how peptide charge and hydrophobicity influence their localization depth within the bilayer, and their dynamic adaptation mechanisms with membrane fluidity, require further exploration.

Beyond delivery and protection functions, liposomes can also effectively improve the sensory attributes of peptide‐based products. Sharma et al. systematically investigated the bitterness masking effect of liposomes on salmon frame protein hydrolysate (SFPH) and plasticized protein (SFPP) (Figure 6e). Liposomes prepared with optimized ultrasonic parameters effectively masked off‐flavors and achieved sustained release [92]. This study represents an expansion of delivery system functionality from “functional carrier” to “sensory modification.” However, the relationship between bitterness masking mechanisms and liposome membrane permeability, as well as its universality for different bitter peptides, requires in‐depth analysis.

Beyond traditional lipid materials, the introduction of novel inorganic materials provides possibilities for constructing smart responsive carriers. Luo et al. used hydroxyapatite (HAP) as a carrier material to construct a proliposome delivery system to improve the delivery efficiency and stability of walnut peptides (WPs) while maintaining their antioxidant activity, and endowed the system with controlled release properties (Figure 6f) [93]. The innovation of this strategy lies in utilizing the rigid structure of inorganic materials to compensate for the mechanical property deficiencies of liposome membranes. However, the degradation behavior of HAP and the potential regulatory effects of calcium ion release on intestinal epithelial cell function require long‐term safety assessment.

In conclusion, through their biomimetic structure and customizable preparation processes, liposomes provide an effective platform for peptide protection, targeted delivery, and release regulation. Current research has confirmed their potential in improving encapsulation efficiency, stability, and functional synergy. It should be noted that liposome size must be carefully optimized in relation to the intestinal mucus barrier. Particles that are too small (<50 nm) may rapidly penetrate the mucus and be cleared, while overly large particles (>500 nm) may become physically entrapped and fail to reach the epithelium. An optimal size range of approximately 100–500 nm balances mucus penetration with sufficient intestinal retention, thereby maximizing peptide absorption [94]. Future research can further explore the compatibility of liposomes with complex food matrices, interactions with gut microbiota, and promote the development of liposomes into intelligent, multi‐functional integrated delivery systems to expand their applications in precision nutrition and health intervention.

### 4.4 Hydrogels

Hydrogels function as oral delivery carriers for peptides primarily by encapsulating bioactive peptides within their hydrophilic three‐dimensional network structures, mimicking the physiological microenvironment, providing excellent biocompatibility, and achieving targeted controlled release through environmentally responsive mechanisms, thereby protecting peptides from gastrointestinal degradation and improving their bioavailability [95, 96]. Hydrogels are typically formed by physical or chemical crosslinking of natural or synthetic polymers and exhibit high water absorption and retention capacity. Among them, hydrogels prepared from natural matrices such as chitosan and sodium alginate offer significant advantages for oral administration due to their high safety and favorable biodegradability [4].

**FIGURE 7:**
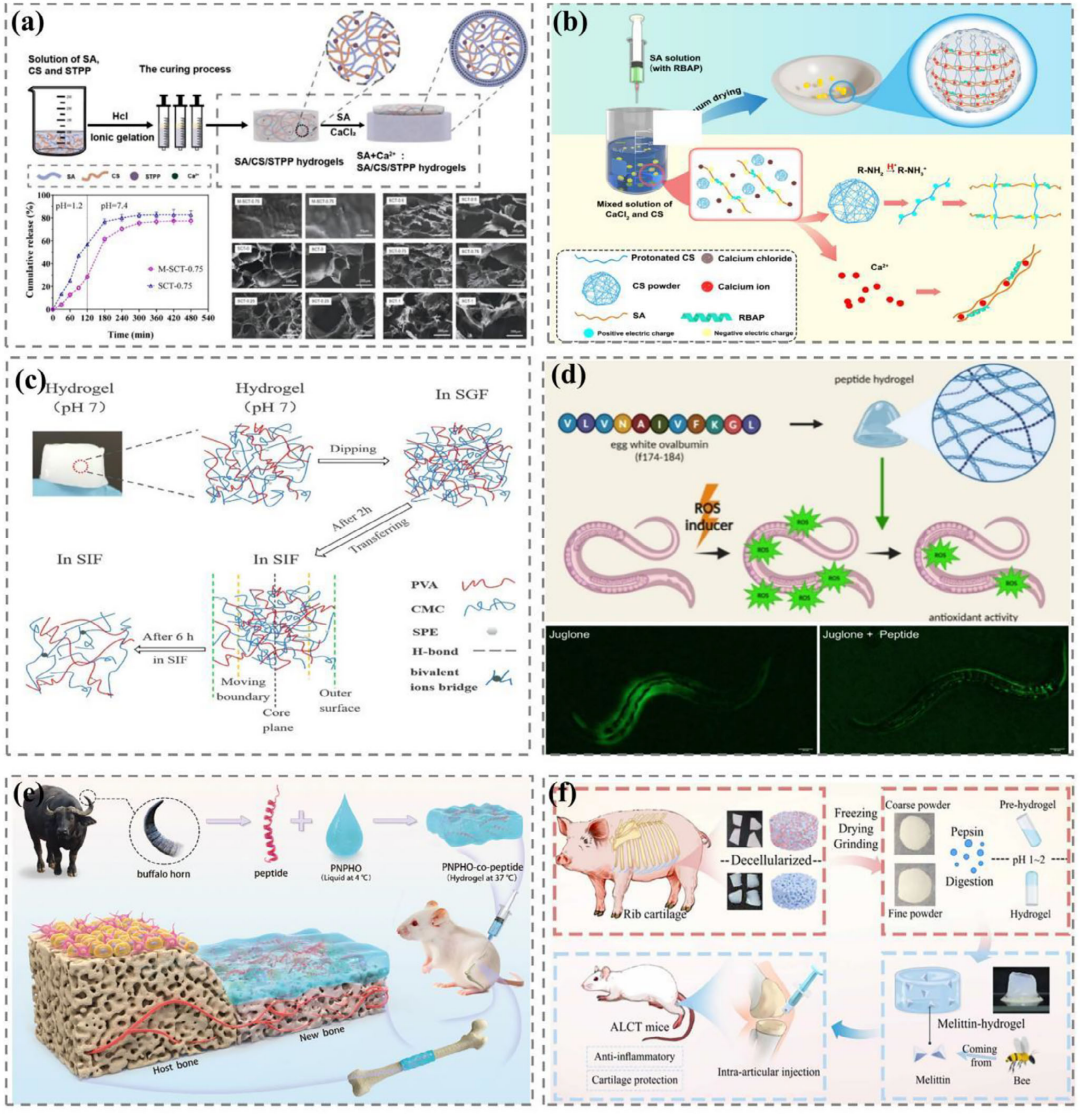
Hydrogel delivery of FBPs. (a) M‐SCT composite hydrogel constructed from chitosan‐sodium alginate‐sodium tripolyphosphate loaded with *Ganoderma lucidum* peptide (GLP). Reproduced with permission [102]. Copyright 2023, MDPI Publishing Group. (b) Chitosan‐alginate hydrogel encapsulating rice bran active peptide (RBAP). Reproduced with permission [18]. Copyright 2023, Elsevier Publishing Group. (c) Carboxymethyl cellulose (CMC)‐polyvinyl alcohol (PVA) hydrogel loaded with soybean peptide (SPE). Reproduced with permission [103]. Copyright 2025, Elsevier Publishing Group. (d) Ovalbumin‐derived peptide VLVNAIVFKGL self‐assembles to form a fibrous network hydrogel. Reproduced with permission [105]. Copyright 2025, American Chemical Society Publishing Group. (e) Bovine horn peptide incorporated into hydrogel to construct a thermosensitive delivery system. Reproduced with permission [106]. Copyright 2024, Wiley Publishing Group. (f) Rib cartilage extracellular matrix (ECM) hydrogel loaded with melittin. Reproduced with permission [107]. Copyright 2025, IOP Publishing Group.

Currently, researchers have developed various hydrogel‐based peptide delivery systems. Polysaccharides represented by chitosan and alginate can construct pH‐responsive smart gels through ionic interactions, and such gels are recognized as promising platforms for oral peptide delivery [4, 97, 98, 99], with the design goal of reducing drug release in the acidic gastric environment while achieving rapid release under intestinal pH conditions [100, 101]. Liu et al. constructed M‐SCT composite hydrogels using chitosan–sodium alginate–sodium tripolyphosphate via ionotropic gelation and layer‐by‐layer deposition techniques, achieving effective loading of *Ganoderma lucidum* peptides (Figure 7a). The three‐dimensional porous structure facilitated the adsorption of peptide molecules, and the electrostatic interactions between the amino groups of chitosan and the carboxyl groups of sodium alginate enhanced the thermal stability and digestive stability of the system. In simulated digestion, less than 30% of the peptides were released in the gastric phase, thereby avoiding premature release, while sustained release was achieved in the intestinal environment, with antioxidant activity being maintained [102]. The innovation of this strategy lies in the construction of a hierarchical structure through layer‐by‐layer assembly, which improves the mechanical strength and environmental responsiveness of the gel. However, the regulatory mechanisms of crosslinking density and pore size distribution on peptide release kinetics, as well as the scalability of the layer‐by‐layer fabrication process, require further investigation.

Similarly, chitosan‐alginate ionically crosslinked hydrogels have also been employed to protect other bioactive peptides. One study utilized chitosan‐sodium alginate hydrogels to load rice bran bioactive peptides (Figure 7b), achieving over 70% sustained release in a neutral intestinal environment and enhancing free radical scavenging capacity by three‐ to seven‐fold, thereby effectively protecting cells from oxidative stress damage and addressing the challenge of low bioavailability [18]. This study demonstrated the dual protective role of hydrogels for peptides, i.e., resisting gastric acid degradation and maintaining sustained biological effects through controlled release. Nevertheless, the matching relationship between the swelling behavior of the hydrogel in the intestinal environment and the peptide release kinetics, as well as the influence of the system on intestinal mucus layer penetration, still requires systematic optimization.

Beyond ion crosslinking strategies, physical hydrogels constructed through freeze‐thaw cycles provide an option for oral peptide delivery without the need for chemical crosslinkers. Ye et al. employed carboxymethyl cellulose and polyvinyl alcohol as matrix materials and fabricated a soybean peptide‐loaded hydrogel delivery system using freeze‐thaw cycling technology (Figure 7c). This system achieved efficient encapsulation of soybean peptides, with minimal release in the gastric phase and complete release in the intestinal phase, and the released peptides retained DPPH and ABTS radical scavenging activities, thus realizing dual optimization of environmentally responsive release and activity preservation [103]. The advantage of this technology lies in avoiding the use of chemical crosslinkers and eliminating potential toxicity concerns. However, the structure‐activity relationships between freeze‐thaw cycle parameters and gel network density, as well as the encapsulation universality for peptides with different molecular weights, still require systematic elucidation.

The functions of hydrogels are not limited to serving as passive carriers; certain active peptide fragments can themselves act as gelators, forming self‐supporting systems with both structural and functional properties [104]. Sunil et al. discovered that the ovalbumin‐derived peptide VLVNAIVFKGL could self‐assemble into a fibrous network hydrogel with good mechanical stability and biocompatibility. Oxidative stress models using *Caenorhabditis elegans* showed that this hydrogel significantly reduced endogenous ROS levels (Figure 7d) [105]. The breakthrough of this study lies in achieving a paradigm shift where the “carrier itself is the active ingredient,” with the peptide serving as both the therapeutic functional unit and a structural component of the gel network. However, the intermolecular interactions driving self‐assembly (e.g., hydrogen bonding and hydrophobic stacking) may induce local conformational changes in the peptide, which could in turn alter its recognition by cell surface receptors or its susceptibility to proteolytic degradation. Therefore, although the self‐assembling peptide hydrogel represents an ideal “carrier‑as‑cargo” platform, systematic structure‑activity relationship studies are still required.

Furthermore, incorporating active peptides as thermosensitive components into gel networks enables the construction of smart implant materials suitable for tissue regeneration. Xue et al. prepared a thermosensitive delivery system by incorporating buffalo horn peptides as active and thermosensitive components into hydrogels (Figure 7e). This hydrogel underwent rapid sol‐gel transition at physiological temperature, forming a three‐dimensional network that effectively filled bone defect areas, promoted osteogenesis and angiogenesis, and exhibited good biodegradability, achieving precise filling and functional regulation to enhance bone regeneration efficiency [106]. The sophistication of this strategy lies in utilizing the intrinsic thermosensitive properties of peptides to achieve unification of gelation and functionality. However, the relationship between peptide thermosensitive transition mechanisms and their sequence structure, as well as the spatiotemporal matching between in vivo degradation rates and new bone formation, requires optimization.

Building upon this foundation, natural matrices derived from biological tissues provide a more ideal microenvironment for constructing biomimetic delivery systems. Yao et al. prepared a biocompatible hydrogel using rib cartilage extracellular matrix (ECM), achieving effective loading of melittin through physical adsorption and molecular interactions (Figure 7f). The system reduced cytotoxicity and achieved sustained release, inhibited phosphorylated ERK/JNK pathway activation, reduced TNFα‐induced apoptosis of ATDC5 cells and inflammatory factor release, and protected cartilage structural integrity. In vivo experiments demonstrated significant reduction in cartilage degeneration progression [107]. The value of this study lies in utilizing natural ECM components to mimic the cartilage microenvironment, providing a “native‐like” delivery matrix for active peptides. However, the compositional complexity and batch variability of ECM pose challenges for mechanistic elucidation and standardized production, and the specific interactions between melittin and various ECM components remain unclear.

In conclusion, with their designable network structures, good biocompatibility, and responsive release capabilities, hydrogels provide an effective and multifunctional delivery platform for peptides. It should be noted, however, that most of the cited in vitro release data were obtained under static buffer conditions (e.g., simulated gastric and intestinal fluids without mechanical agitation or flow), which do not fully recapitulate the dynamic physiological environment of the gastrointestinal tract, including peristaltic shear forces, variable fluid volumes, and real‐time enzyme secretion. Consequently, the reported release profiles may overestimate intestinal retention or underestimate burst release. Future studies should adopt more biorelevant models, such as dynamic gastrointestinal simulators or microfluidic gut‐on‐a‐chip systems, to better predict in vivo performance. Existing research has demonstrated their application potential in oral delivery, antioxidant protection, and tissue repair. Future research can further focus on developing more intelligent responsive systems, exploring multi‐peptide co‐delivery mechanisms, elucidating dynamic interactions between hydrogels and biological barriers, and promoting their practical development in clinical treatment and functional food applications.

### 4.5 Composite Systems

Composite delivery systems integrate the structural characteristics and functional advantages of multiple single carriers to construct synergistic delivery networks, exhibiting significant potential in improving peptide stability, controlled release performance, and bioavailability. These systems typically integrate physical encapsulation, environmental responsiveness, and targeted delivery through multi‐scale compounding of materials such as lipids, polysaccharides, and proteins via strategies including emulsion gelation, surface coating, and core‐shell construction.

**FIGURE 8:**
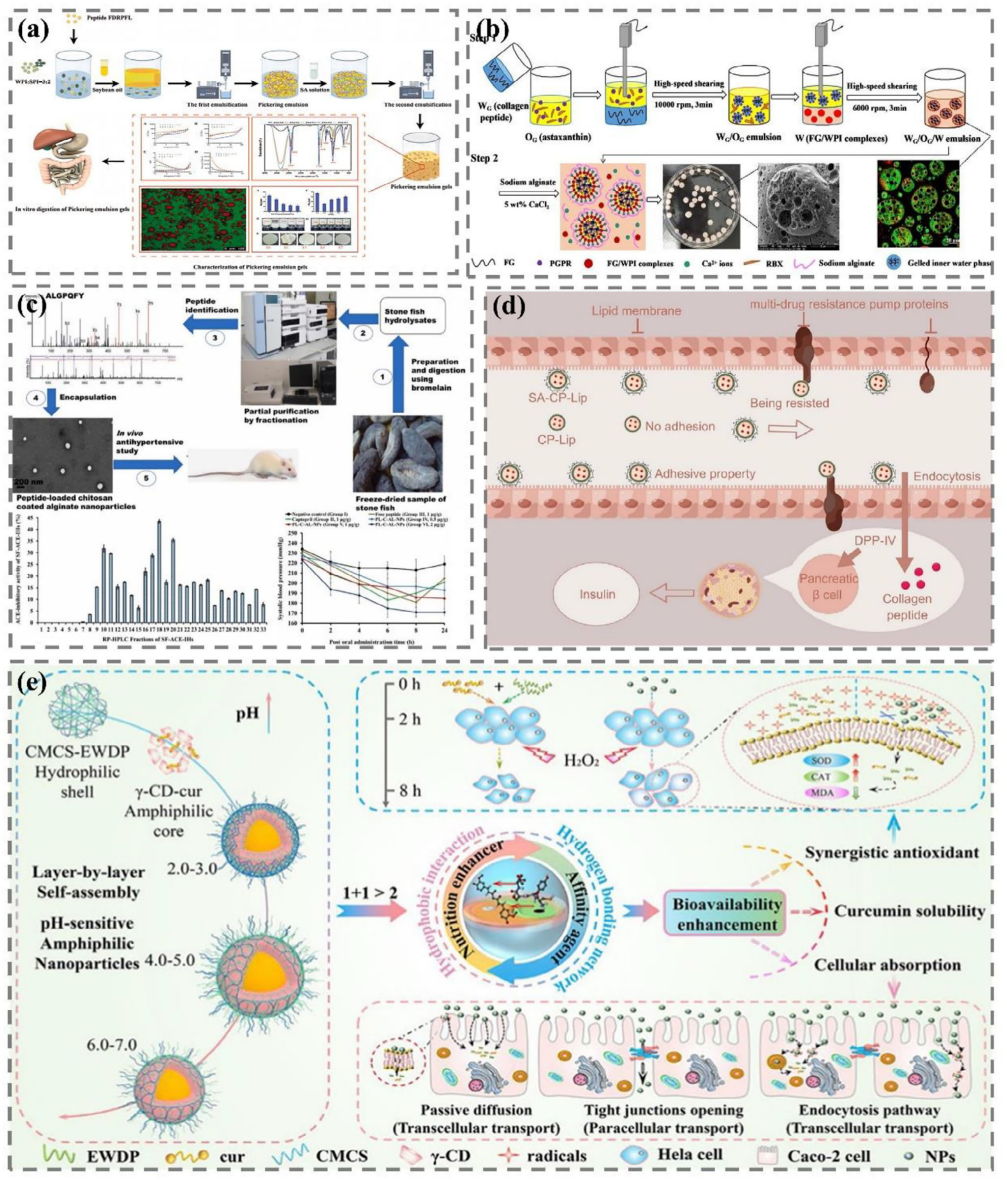
Delivery of FBPs by composite systems. (a) Whey protein isolate (WPI)/soy protein isolate (SPI) composite particles stabilize Pickering emulsion (PE) and add sodium alginate (SA) to form Pickering emulsion gel (PEG) for encapsulating peptide FDRPFL. Reproduced with permission [108]. Copyright 2024, Elsevier Publishing Group. (b) Water‐in‐oil emulsion‐filled hydrogel beads constructed from rice bran wax and W/O emulsion for co‐delivery of collagen peptides and astaxanthin. Reproduced with permission [109]. Copyright 2023, Elsevier Publishing Group. (c) Sodium tripolyphosphate (TPP)‐crosslinked chitosan‐coated alginate nanoparticles (PL‐C‐AL‐NP) for encapsulating stonefish‐derived ACE inhibitory peptide ALGPQFY. Reproduced with permission [110]. Copyright 2024, Elsevier Publishing Group. (d) Sodium alginate‐coated liposomes delivering DPP‐IV inhibitory collagen peptides. Reproduced with permission [19]. Copyright 2023, Elsevier Publishing Group. (e) A composite nanoparticle delivery system with carboxymethyl chitosan (CMCS) as the shell and γ‐cyclodextrin (γ‐CD) as the core for co‐delivery of egg white peptide EWDP and curcumin. Reproduced with permission [111]. Copyright 2022, American Chemical Society Publishing Group.

Researchers have developed various composite systems for peptide delivery. The combination of emulsions with gel networks can achieve dual synergistic effects in micro‐interface stabilization and macro‐structural support. Sun et al. stabilized Pickering emulsions (PE) using whey protein isolate (WPI)/soy protein isolate (SPI) composite particles and incorporated sodium alginate (SA) to form Pickering emulsion gels (PEG), achieving efficient encapsulation of the peptide FDRPFL with super Case II diffusion release, which significantly improved its bioaccessibility and storage stability (Figure 8a) [108]. The innovation of this strategy lies in combining interfacial adsorption with three‐dimensional gel networks to construct a delivery platform with high loading capacity and environmental responsiveness. However, whether the introduction of gel networks alters the matching between peptide release kinetics in the intestine and absorption sites, as well as its impact on mucus penetration behavior, requires further investigation. Similarly, Wei et al. utilized rice bran wax and W/O emulsions to construct water‐in‐oil emulsion‐filled hydrogel beads for the co‐delivery of collagen peptides and astaxanthin (Figure 8b). The addition of rice bran improved storage stability, delayed release rates, and enhanced bioaccessibility [109]. This study suggests that wax components not only serve as structural enhancers but their crystallization characteristics may also influence peptide distribution and release behavior at the oil‐water interface. However, the impact of wax source variations and their molecular interaction mechanisms with emulsion interfaces requires systematic elucidation.

Beyond physical structure compounding, combining polysaccharides with nanocarriers at the molecular level can further enhance the environmental adaptability and functional targeting of delivery systems. Auwal et al. cross‐linked chitosan with sodium tripolyphosphate (TPP) to construct chitosan‐coated alginate nanoparticles (PL‐C‐AL‐NP) for encapsulating the ACE inhibitory peptide ALGPQFY derived from stonefish (Figure 8c). This system demonstrated significant systolic blood pressure reduction in hypertensive models, with effects superior to free peptides, validating its clinical translation potential [110]. The breakthrough of this study lies in constructing multi‐layered protective barriers through layer‐by‐layer polysaccharide assembly, effectively protecting peptides against gastric acid and enzymatic degradation. However, the regulatory mechanisms of multilayer thickness and compactness on peptide release kinetics, as well as potential mucus trapping effects induced by the cationic nature of chitosan, require optimization and trade‐off considerations.

Furthermore, polysaccharide coating can specifically improve the stability and cellular uptake efficiency of traditional carriers such as liposomes. Wu et al. employed sodium alginate‐coated liposomes to deliver DPP‐IV inhibitory collagen peptides (Figure 8d). SA coating significantly enhanced carrier stability and promoted the uptake of peptide‐loaded liposomes, thereby increasing peptide absorption efficiency [19]. This strategy represents “functional modification” rather than simple “physical wrapping” of liposomes by polysaccharides. However, the regulatory effects of alginate molecular weight and M/G ratio on coating layer compactness and liposome surface charge, as well as their selective influence on intestinal epithelial cell uptake pathways, require in‐depth analysis.

At a more refined scale, designing core‐shell structures with distinct functional zones provides an ideal platform for the co‐delivery of active substances with different properties. Yang et al. innovatively constructed an amphiphilic nanoparticle delivery system using carboxymethyl chitosan (CMCS) as the shell and γ‐cyclodextrin (γ‐CD) as the core, achieving co‐delivery of hydrophilic egg white peptides (EWDP) and hydrophobic curcumin (Figure 8e). CMCS and EWDP acted synergistically through hydrogen bonding networks and hydrophobic interactions, significantly improving overall antioxidant activity, bioaccessibility, and cellular uptake performance [111]. The sophistication of this design lies in utilizing cyclodextrin cavities to encapsulate hydrophobic molecules while employing chitosan derivative hydrophilic chains to complex peptides, achieving “compartmentalized loading with synergistic delivery.” However, whether the release ratios and sequences of the two active components can be independently regulated, and whether their intracellular dissociation and transport pathways interfere with each other, requires further investigation.

Through multi‐dimensional synergy of materials and structures, composite delivery systems overcome the functional limitations of single carriers, providing powerful tools for achieving efficient and intelligent peptide delivery. Current research has preliminarily validated their value in controlled release, efficacy enhancement, and targeting. Future studies need to further explore their scalable manufacturing processes, in vivo metabolic fate, and clinical translation pathways, while deeply investigating the dynamic interaction mechanisms between composite material interfaces and biological barriers. This will advance functional peptide products from “structural replication” toward “functional design,” ultimately realizing their practical applications in precision nutrition and health intervention.

In summary, the diversity of delivery systems – including nanoparticles, nanoemulsions, liposomes, hydrogels, and composite platforms – provides a versatile toolbox for addressing the multifaceted barriers to oral peptide delivery. Each carrier type offers distinct advantages in encapsulation, environmental responsiveness, and targeted release, yet no single system is universally optimal. Given the considerable variation among FBPs in effective doses, molecular weight, charge distribution, hydrophobicity, and susceptibility to gastrointestinal degradation, the rational selection of a delivery carrier must be guided by the specific absorption requirements and pharmacokinetic profile of the peptide cargo. A peptide requiring prolonged systemic circulation may benefit from the sustained‐release properties of hydrogels, whereas one demanding rapid transepithelial transport may be better served by lipid‐based nanoparticles or nanoemulsions. The ultimate goal is to achieve a precise match between carrier functionality and peptide absorption efficacy, thereby maximizing the proportion of the administered dose that reaches the target site in its bioactive form. Future efforts should move beyond empirical carrier screening toward systematic structure‐property‐activity correlations, enabling the predictive design of delivery systems that are tailored to the unique physicochemical and pharmacological characteristics of individual FBPs.

## 5 Biological Function

**FIGURE 9:**
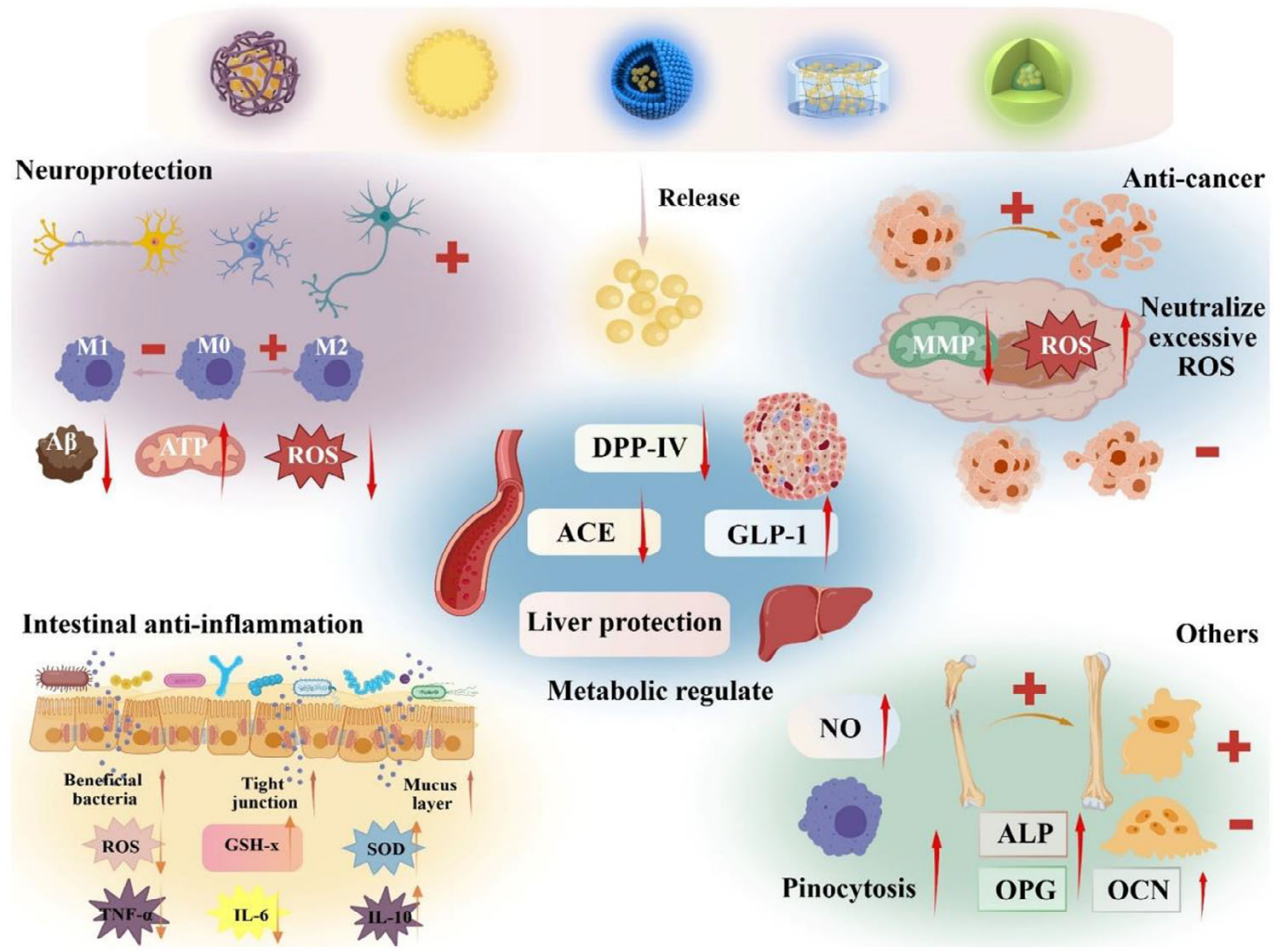
Multidimensional biological activities and mechanisms of action exerted by bioactive peptide‐loaded delivery systems after peptide release. Created with BioGDP.com [147].

FBPs are short‐chain amino acid fragments derived from natural foods, which possess unique advantages such as low molecular weight and high bioactivity. Due to their advantages, FBPs exhibit a range of biological functions in physiological regulation, including but not limited to neural signal modulation, inflammatory response regulation, metabolic homeostasis maintenance, and orderly cell cycle progression. Their intrinsic low toxicity, high tissue permeability, and biodegradability confer distinct advantages for applications in dietary intervention and functional foods, particularly in supporting nervous system function, modulating the inflammatory microenvironment, optimizing energy metabolism, and regulating cell proliferation and differentiation. However, bottlenecks including low oral bioavailability and susceptibility to degradation in the gastrointestinal tract limit their functional exertion. Therefore, developing efficient delivery carriers such as nanoparticles, nanoemulsions, liposomes, and hydrogels to protect the active structure, enhance absorption, and achieve targeted delivery has become a key strategy to unlock their maximum therapeutic potential and promote their practical application in chronic disease management and functional foods (Figure 9).

### 5.1 Neuroprotection

FBPs demonstrate significant biological potential in supporting neurological function. Alzheimer's disease (AD) and spinal cord injury (SCI) represent two major categories of neurodegenerative and injury‐related conditions, characterized by complex pathological processes involving abnormal protein aggregation, neuroinflammation, energy metabolic dysregulation, and disruption of the regenerative microenvironment [112]. FBPs exert multi‐target neuroprotective effects through mechanisms including inhibition of Aβ aggregation, attenuation of inflammatory responses, protection of mitochondrial function, and promotion of nerve regeneration [113]. However, the inherent susceptibility of FBPs to enzymatic degradation and their limited ability to effectively cross the blood‐brain barrier (BBB) or blood‐spinal cord barrier (BSCB) necessitate the development of advanced delivery systems to achieve precise and efficient targeted intervention.

**FIGURE 10:**
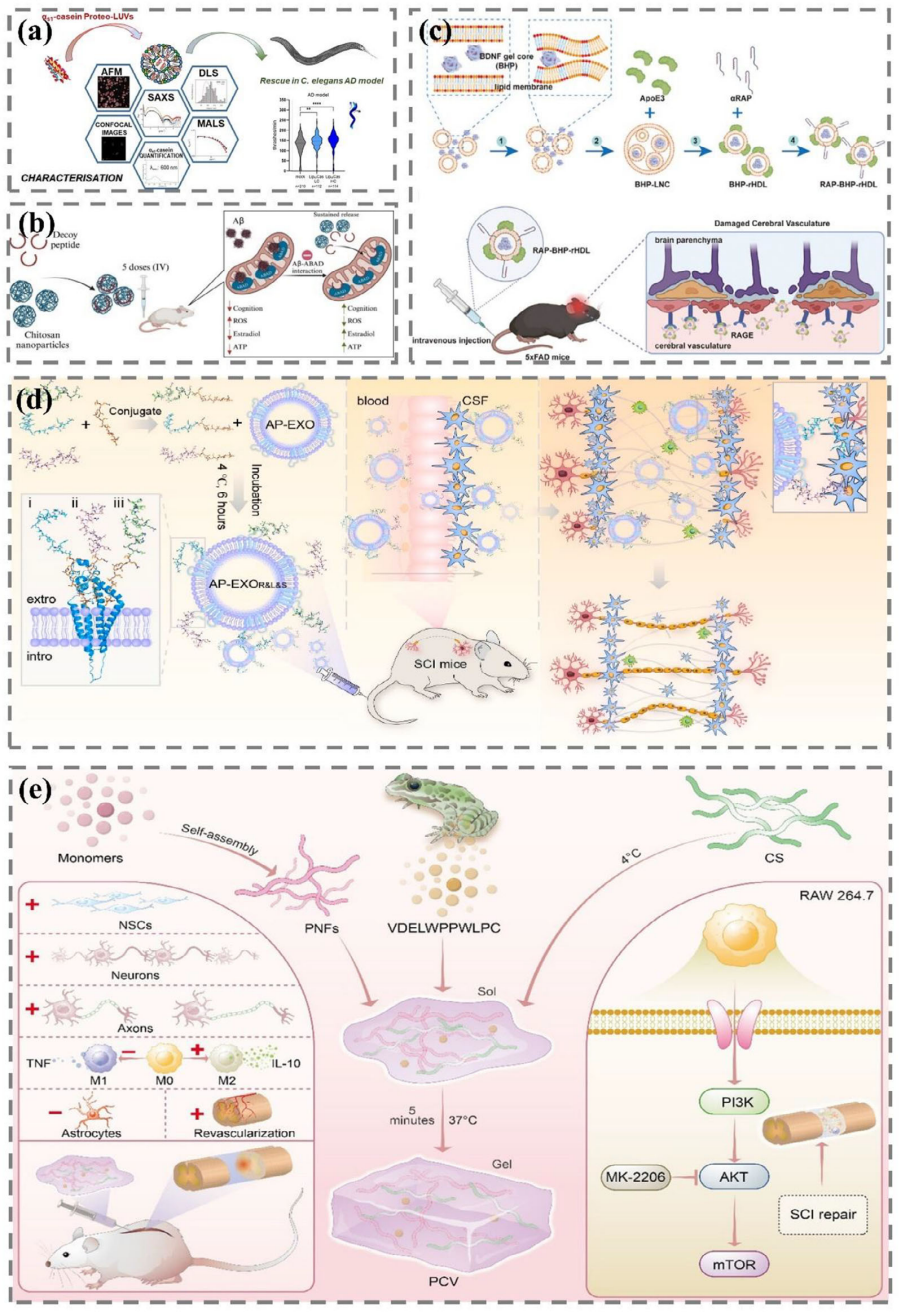
FBPs for neuroprotection. (a) Milk αs1‐casein liposomes (Proteo‐LUV) enable targeted enrichment of peptides at AD pathological sites and effectively inhibit Aβ toxicity at extremely low doses. Reproduced with permission [114]. Copyright 2023, American Chemical Society Publishing Group. (b) Specific decoy peptides (DP) encapsulated in polymeric chitosan nanoparticles (CSNP) achieve brain targeting, significantly improve neuroinflammation, reduce Aβ deposition, enhance antioxidant activity, and realize neuronal protection. Reproduced with permission [11]. Copyright 2025, Elsevier Publishing Group. (c) Lipoprotein biomimetic nanocarriers (RAP‐BHP‐rHDL) fuse with liposomes to encapsulate and target‐deliver brain‐derived neurotrophic factor (BDNF), which systematically protects nerves by alleviating nerve damage, promoting neurogenesis, and remodeling brain microvascular structure. Reproduced with permission [115]. Copyright 2025, Wiley Publishing Group. (d) Plasma exosomes (AP‐EXO) co‐load neuron‐targeting peptide RVG and dual‐effect growth‐promoting peptides (ILP/ISP), achieving a dual breakthrough in neuroprotection and repair. Reproduced with permission [116]. Copyright 2023, Elsevier Publishing Group. (e) Amphibian‐derived nerve regeneration peptide VD11 (VDELWPPWLPC) is combined with bioactive peptide nanofibers (PNF)/chitosan (CS) to construct a hydrogel, forming a dual mechanism of anti‐inflammation and pro‐angiogenesis to achieve neuroprotective effects. Reproduced with permission [117]. Copyright 2025, Wiley Publishing Group.

Targeted intervention against Aβ pathology represents a core strategy for inhibiting toxic protein aggregation. Paterna et al. constructed a milk αs1‐casein‐based proteoliposome (Proteo‐LUV) nanosystem (Figure 10a). This system not only achieved targeted enrichment of the peptide at AD pathological sites but also exhibited excellent neuroprotective efficacy. Notably, Proteo‐LUV effectively inhibited Aβ toxicity at extremely low doses while completely avoiding the potential cytotoxicity associated with free αs1‐casein peptide through a spatial isolation strategy [114]. This design approach offers new insights for enhancing the safety profile of FBP‐based applications. Furthermore, intervening in downstream toxic pathways of Aβ can synergistically enhance neuroprotective effects. Youssef et al. encapsulated specific decoy peptides (DP) into chitosan nanoparticles (CSNP) (Figure 10b), constructing a targeted delivery system with high BBB penetration efficiency [11]. This system demonstrated superior brain enrichment capability and significantly reduced off‐target effects. Compared with free DP, the CSNP‐loaded system not only significantly reversed cognitive impairment in a neuroinflammation mouse model by specifically blocking the formation of the toxic Aβ‐ABAD complex, but also effectively reduced Aβ deposition. Concurrently, it restored mitochondrial ATP synthesis capacity and SOD antioxidant activity, thereby reshaping neuronal energy metabolic homeostasis. This strategy suggests that targeted delivery systems can function not merely as carriers but can amplify the multi‐target functions of FBPs through synergistic regulation of multiple pathological pathways.

Regarding the promotion of nerve regeneration and repair, targeted delivery of neurotrophic factors or activation of endogenous repair programs represents a critical approach. Wang et al. innovatively constructed a membrane bud‐inspired apolipoprotein biomimetic nanocarrier (RAP‐BHP‐rHDL) and fused it with liposomes to achieve efficient encapsulation and targeted delivery of brain‐derived neurotrophic factor (BDNF) (Figure 10c). Dual‐functional modification of this nanocarrier with apolipoprotein E3 and the αRAP peptide significantly enhanced its BBB penetration capability, enabling precise anchoring to damaged cerebrovascular regions in AD model mice. By alleviating neuronal injury, promoting neurogenesis, and remodeling cerebral microvascular structure, this system systematically repaired the function of the neurovascular unit, ultimately leading to significant recovery of memory and cognitive abilities in 5×FAD mice [115]. The innovation of this study lies not only in the delivery of a single factor but also in the biomimetic design that mimics natural repair processes, providing a new paradigm for intervention in complex neurological disorders.

Similarly, activating endogenous regenerative capacity shows substantial potential in spinal cord injury repair. Ran et al. constructed an intelligent biological scaffold based on autologous plasma exosomes (AP‐EXO) by precisely co‐loading the neuron‐targeting peptide RVG and dual‐effect growth‐promoting peptides (ILP/ISP) (Figure 10d), achieving dual breakthroughs in neuroprotection and repair. This system could specifically accumulate in the spinal cord injury area and synergistically release ILP/ISP peptide chains to activate endogenous nerve regeneration programs, thereby reconstructing spinal neural circuitry and significantly restoring motor function in mice [116]. Notably, peptide‐loaded human plasma exosomes (HP‐EXO) exhibited excellent safety advantages in a nude mouse model of spinal cord injury, providing important support for subsequent clinical translation. However, challenges regarding large‐scale production and batch consistency of exosomes remain, necessitating further optimization of preparation processes.

Furthermore, constructing a biomimetic microenvironment can further support neural structure regeneration. Sun et al. compounded the amphibian‐derived nerve regeneration peptide VD11 with bioactive peptide nanofibers (PNF)/chitosan (CS) (Figure 10e) to form a PCV intelligent responsive hydrogel scaffold with a three‐dimensional porous structure. Through synergistic effects between peptides and the matrix, this scaffold successfully established a nerve regeneration microenvironment, activating the MAPK/ERK signaling pathway to promote directional differentiation of neural stem cells into neurons and guide axonal directional extension. Simultaneously, it regulated the polarization balance of M1/M2 macrophages and inhibited excessive astrocyte activation, forming a dual protective mechanism involving anti‐inflammation and pro‐angiogenesis. Moreover, the hydrogel built a biological bridge for axon regeneration by enhancing local revascularization and endogenous neural stem cell homing, while inhibiting glial scar formation to reduce immune rejection, ultimately increasing the recovery efficiency of motor function in injured spinal cords by 42% [117]. This study emphasizes the critical role of biomimetic material design in regulating cellular behavior and the microenvironment. However, it also suggests that maintaining compatibility between hydrogel degradation rate and functional peptide release kinetics in complex in vivo environments requires further investigation.

In summary, targeted delivery and controlled release of FBPs via delivery systems have become effective strategies for intervening in neurodegenerative conditions and promoting injury repair. Current research has made important progress in carrier design, mechanistic exploration, and animal model validation. However, several aspects warrant further attention: first, the long‐term biosafety of carriers and their potential impact on the functional activity of FBPs; second, the feasibility of clinical translation from animal models, including large‐scale production and quality control; and third, the synergistic mechanisms between different FBPs and between FBPs and carrier materials require deeper elucidation. Future research should focus on optimizing delivery efficiency while thoroughly investigating the multi‐target regulatory mechanisms of FBPs within the neurovascular unit, immune microenvironment, and metabolic networks, thereby advancing this field toward practical applications.

### 5.2 Intestinal Anti‐inflammation

Due to their low molecular weight, high biocompatibility, and strong structural modifiability, FBPs exhibit unique potential in the regulation of inflammation‐related physiological processes. Chronic intestinal inflammation, exemplified by ulcerative colitis (UC), is pathologically characterized by a vicious cycle involving “intestinal barrier damage, dysbiosis, and excessive immune activation” [118]. FBPs can intervene in this cycle through multiple mechanisms, including epithelial barrier repair, intestinal microecology regulation, and direct anti‐inflammatory effects. However, FBPs are susceptible to inactivation in the complex gastrointestinal environment and face challenges in targeting lesioned sites. Therefore, the utilization of delivery systems to achieve their protection, enrichment, and controlled release is crucial for enhancing their physiological regulatory functions and reducing systemic exposure risks.

**FIGURE 11:**
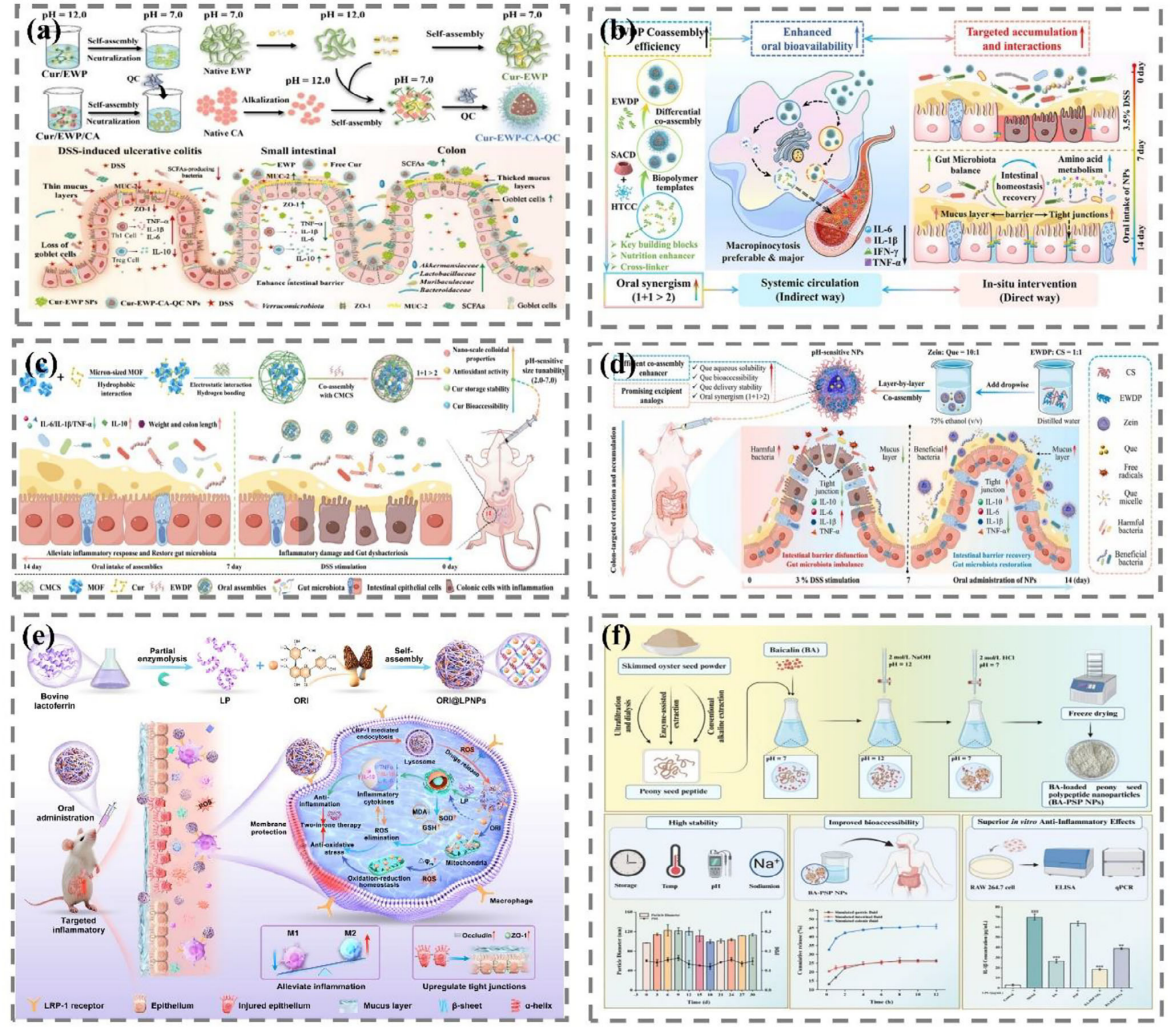
FBPs improve intestinal inflammation. (a) Curcumin‐egg white peptide‐casein‐quaternized chitosan nanoparticles (Cur‐EWP‐CA‐QC NPs) promote the trans‐intestinal epithelial transport of Cur/EWP, upregulate the expression of tight junction proteins (zonula occludens‐1, occludin) to strengthen the physical barrier; continuously regulate the gut microbiota metabolic axis, inhibit the excessive activation of the NF‐κB pathway, thereby exerting a multi‐target anti‐inflammatory effect. Reproduced with permission [119]. Copyright 2024, Elsevier Publishing Group. (b) The nano‐delivery system with quaternized chitosan (HTCC) as the shell and succinic acid‐modified γ‐cyclodextrin (SACD) as the core co‐delivers egg white‐derived peptides (EWDP) and curcumin (Cur). It has colon‐targeting properties and sustainable release capability, and can significantly improve cell uptake efficiency and oral bioavailability through enhancing mucus penetration and macropinocytosis‐mediated transcellular transport, thereby effectively regulating systemic inflammatory responses. Reproduced with permission [120]. Copyright 2025, American Chemical Society Publishing Group. (c) Carboxymethyl chitosan (CMCS) and γ‐cyclodextrin metal‐organic framework (MOF) co‐deliver egg white‐derived peptides (EWDP) and curcumin. By enhancing mucosal barrier zinc ion homeostasis and goblet cell mucin secretion, a closed‐loop regulation of “anti‐inflammation‐promoting repair” is formed. Reproduced with permission [121]. Copyright 2024, American Chemical Society Publishing Group. (d) The colon‐targeted nano‐delivery system co‐assembled by protein‐derived peptides (EWDP) and chondroitin sulfate (CS) synergistically delivers EWDP and quercetin (Que). After EWDP accumulates in a colon‐targeted manner, it significantly repairs the intestinal barrier and restores the balance of the intestinal microbiota, thereby systematically improving the intestinal microenvironment and immune homeostasis. Reproduced with permission [122]. Copyright 2025, Elsevier Publishing Group. (e) Lactoferrin peptide (LP) self‐assembles with flavonoid orientin (ORI) into nanoparticles (ORI@LPNPs). This system blocks the cell apoptosis cascade by inhibiting mitochondrial membrane potential collapse and restoring GSH/GSSG redox balance; by regulating the TLR4/MyD88/NF‐κB signaling pathway, it significantly reduces the expression level of pro‐inflammatory factors, achieving precise anti‐inflammation. Reproduced with permission [123]. Copyright 2025, Elsevier Publishing Group. (f) The peony seed peptide‐based delivery system encapsulates baicalin (BA), which, on the one hand, inhibits the expression and secretion of pro‐inflammatory factors; on the other hand, promotes the production of anti‐inflammatory factors, achieving inflammatory balance. Reproduced with permission [124]. Copyright 2025, Elsevier Publishing Group.

In response to the aforementioned pathological links, researchers have developed various intelligent delivery strategies. Regarding barrier repair and microbiota regulation, the design of delivery systems emphasizes integrated intervention within the intestinal microenvironment. Li et al. constructed a curcumin‐egg white peptide‐casein‐quaternized chitosan nanoparticle (Cur‐EWP‐CA‐QC NPs) delivery system based on a non‐covalent dynamic soft assembly strategy involving egg white peptides and curcumin (Cur) (Figure 11a). Leveraging nanoscale stability and enhanced mucoadhesive properties, Cur‐EWP‐CA‐QC NPs not only promote the trans‐epithelial transport of Cur/EWP and upregulate tight junction protein expression (zonula occludens‐1, occludin) to strengthen the physical barrier, but also enable unabsorbed components to continuously modulate the intestinal flora metabolic axis and inhibit excessive NF‐κB pathway activation, thereby exerting multi‐target anti‐inflammatory effects [119]. This strategy suggests that delivery system design should consider not only the encapsulation and release of active ingredients but also their dynamic interactions with the intestinal microenvironment.

Furthermore, enhancing delivery efficiency and targeting specificity represents a core direction for optimizing intervention outcomes. Yang et al. employed quaternized chitosan (HTCC) as the shell and succinic acid‐modified γ‐cyclodextrin (SACD) as the core to achieve efficient encapsulation and oral co‐delivery of egg white‐derived peptides (EWDP) and curcumin (Cur) (Figure 11b). This carrier exhibits colon‐targeting properties and sustained‐release capabilities, significantly improving cellular uptake efficiency and oral bioavailability by enhancing mucus penetration and macropinocytosis‐mediated transcellular transport. Its functional mechanism involves bidirectional regulation: upregulating tight junction protein expression to reduce mucosal permeability and repair the intestinal barrier, while increasing the abundance of short‐chain fatty acid‐producing bacteria to reshape microbiota structure and maintain intestinal metabolic homeostasis [120]. The innovation of this study lies in simultaneously addressing the gastrointestinal stability and targeted absorption challenges of peptides through rational carrier material design. However, whether macropinocytosis activation exhibits cell‐type specificity and its potential impact on intestinal immune cells requires further investigation.

Through the synergistic effects of active ingredients and functional mimicry of peptides, more refined regulatory networks can be constructed. Zhang et al. developed a delivery system using carboxymethyl chitosan (CMCS) and γ‐cyclodextrin metal‐organic frameworks (MOF) as building blocks, similarly achieving co‐loading and intestinal targeted delivery of EWDP and curcumin (Figure 11c). Oral administration of EWDP combined with curcumin synergistically inhibited NF‐κB phosphorylation and NOD‐like receptor family pyrin domain containing 3 (NLRP3) inflammasome activation, significantly reducing pro‐inflammatory factor expression (IL‐6, TNF‐α). Additionally, it selectively enriched short‐chain fatty acid‐producing strains such as *Akkermansia muciniphila*, restoring microbiota α‐diversity to healthy levels. Notably, the amino acid sequence specificity of EWDP enables it to mimic host defense peptide functions, forming a closed‐loop “anti‐inflammatory‐pro‐repair” regulatory network by enhancing mucosal barrier zinc homeostasis and goblet cell mucin secretion [121]. Notably, compared with free EWP/EWDP, free Cur, their physical mixture, and blank carriers, the nanodelivery systems loaded with peptides and Cur exhibit significantly enhanced activities in intestinal stability, transepithelial transport efficiency, barrier repair, and microbiota modulation. Free active substances are prone to degradation after oral administration and can hardly reach the colon, whereas the delivery systems substantially increase the target‐site enrichment concentration and synergistic effect, directly confirming that the carrier is an essential prerequisite for achieving oral multi‐target anti‐inflammatory regulation, rather than a passive delivery vehicle. The above strategies achieve multi‐step intervention in intestinal inflammation through the co‐delivery of peptides and anti‐inflammatory active molecules, providing a reference for the design of oral peptide‐based anti‐inflammatory preparations.

Utilizing the self‐assembly properties of peptides to construct multifunctional carriers represents another effective approach. Ma et al. constructed a colon‐targeted nano‐delivery system based on the co‐assembly of egg white‐derived peptides (EWDP) and chondroitin sulfate (CS), achieving synergistic delivery of EWDP and quercetin (Que) (Figure 11d). Here, EWDP not only acts as a co‐assembly enhancer, improving carrier stability and Que water solubility, but also significantly repairs the intestinal barrier (tight junctions and mucus layer) after colon‐targeted accumulation and restores intestinal microbiota balance (e.g., enriching *Lachnospiraceae* and eliminating *Bacteroidota*), thereby systematically improving the intestinal microenvironment and immune homeostasis [122]. This study highlights the dual function of peptides as both assembly units and active ingredients. However, whether conformational changes of peptides during co‐assembly affect subsequent biological activity, and the spatiotemporal matching of carrier disassembly, peptide release kinetics, and functional expression, remain issues requiring in‐depth investigation.

In contrast to systemic microenvironmental regulation, another category of strategies focuses on precise inhibition of specific intracellular anti‐inflammatory signaling pathways. Ran et al. utilized amphiphilic lactoferrin peptides (LP) to self‐assemble with the natural flavonoid orientin (ORI), forming multifunctional spherical nanoparticles (ORI@LPNPs) with ROS‐responsive properties, thereby constructing a precision anti‐inflammatory platform (Figure 11e). This system exhibits excellent stability in the gastric environment, with its core mechanism involving specific recognition of low‐density lipoprotein receptor‐related protein 1 (LRP‐1) by the LP peptide segment, achieving targeted delivery to activated macrophages (cellular uptake efficiency of 93.83%). On one hand, the system blocks apoptotic cascades by inhibiting mitochondrial membrane potential collapse and restoring GSH/GSSG redox balance; on the other hand, it significantly reduces pro‐inflammatory factor expression levels (IL‐1β, IL‐6, TNF‐α) by regulating the Toll‐like receptor 4 (TLR4)/MyD88/NF‐κB signaling pathway. Oral administration of ORI@LPNPs increased drug enrichment in inflamed intestinal segments by 2.26‐fold, with its mucoadhesive properties benefiting from the intestinal permeability‐enhancing effect of LP [123]. Notably, this nanosystem completely preserves the biosafety of food‐derived bioactive substances. The breakthrough of this study lies in transitioning from passive delivery to active targeting. However, whether LRP‐1 activation might interfere with other physiological functions of this receptor requires long‐term safety assessment.

Furthermore, peptide‐based nanocarriers themselves may actively participate in inflammation regulation. Chen et al. developed a pH‐driven self‐assembled nano‐delivery system based on peony seed peptides for efficient encapsulation of baicalin (BA) (Figure 11f). These nanoparticles significantly improved the oral stability and bioavailability of BA. Functional studies demonstrated that this nanosystem inhibits pro‐inflammatory factor gene expression and secretion (IL‐1β, IL‐6, TNF‐α) while promoting anti‐inflammatory factor IL‐10 production, thereby reshaping inflammatory balance at both transcriptional and protein levels [124]. These results indicate that peptide‐based nanocarriers not only enhance active ingredient delivery efficiency through physical protection but may also actively participate in regulating key inflammatory pathways through synergistic effects with encapsulated components. However, distinguishing the respective contributions of the nanocarrier itself, peptide fragments released after carrier disassembly, and the encapsulated active ingredients, as well as understanding their synergistic mechanisms, remains a complex issue requiring future investigation.

It should be noted, however, that most of the cited studies reporting gut microbiota modulation by FBPs have not distinguished whether the observed effects stem from direct interactions of intact peptides with microbial cells or from indirect actions via microbial metabolites generated from peptide degradation. Recent reviews have highlighted that both pathways may coexist: peptides can serve as nitrogen/carbon sources for gut microbes, yielding short‐chain fatty acids and other fermentation products that indirectly influence microbial ecology, whereas certain intact peptides may also directly promote or inhibit specific bacterial strains through oligopeptide permease (Opp) and di‐/tripeptide permease (Dpp) systems [125, 126]. Future studies should employ targeted approaches such as non‐degradable peptide analogs, germ‐free animal models, or ex vivo microbiota cultures to delineate the relative contributions of these two mechanisms.

In conclusion, through sophisticated delivery system design, FBPs can achieve synergistic intervention across multiple pathological aspects of ulcerative colitis. Current research has made substantial progress in carrier construction, mechanism elucidation, and animal model validation. However, several issues warrant attention: first, deepening the understanding of carrier‐host interaction mechanisms, particularly with the intestinal immune system and microbiota; second, systematically conducting long‐term safety assessments and clinical translation studies, including scalable carrier preparation, quality control, and in vivo fate and function in humans; and third, exploring precision delivery strategies based on individual intestinal microecological characteristics. In addition, it should also be noted that many of the delivery systems discussed above employ polysaccharide‐based materials – such as chitosan, cyclodextrin, and chondroitin sulfate – which may themselves serve as fermentable substrates for gut microbiota. Consequently, the observed improvements in microbial composition and short‐chain fatty acid production could partly originate from the prebiotic activity of the carrier rather than solely from the encapsulated peptide or polyphenol. Distinguishing the carrier‐derived effects from the peptide‐specific effects remains a challenge, as most studies did not include carrier‐only controls in microbiota analyses. Future work should therefore incorporate such controls and systematically evaluate how the chemical structure, degree of polymerization, and degradation rate of polysaccharide carriers influence their interactions with the gut ecosystem. This will be essential for accurately attributing the anti‐inflammatory benefits and for guiding the rational design of delivery systems that combine efficient transport with desirable prebiotic functionality. Future research should optimize delivery efficiency while thoroughly investigating the synergistic regulatory networks between FBPs and carrier materials, advancing these intelligent delivery strategies toward personalized precision nutrition.

### 5.3 Metabolic Regulation

Based on their sequence diversity and receptor affinity, FBPs exhibit distinct biological functions in the regulation of metabolic homeostasis. Metabolic dysregulation, represented by type 2 diabetes and hypertension, involves multiple pathological features including insulin resistance, overactivation of the renin‐angiotensin system (RAS), and gut microbiota dysbiosis, collectively leading to impaired glucose and blood pressure homeostasis as well as multi‐organ dysfunction. FBPs exert their functions through various mechanisms, including DPP‐IV inhibition to maintain GLP‐1 levels, ACE blockade to modulate vascular tone, or regulation of the calcium‐sensing receptor (CaSR) [127]. However, FBPs are susceptible to enzymatic degradation during oral delivery, resulting in low bioavailability. Therefore, achieving activity protection and precise regulation through delivery systems is crucial for fully realizing their metabolic regulatory potential.

**FIGURE 12:**
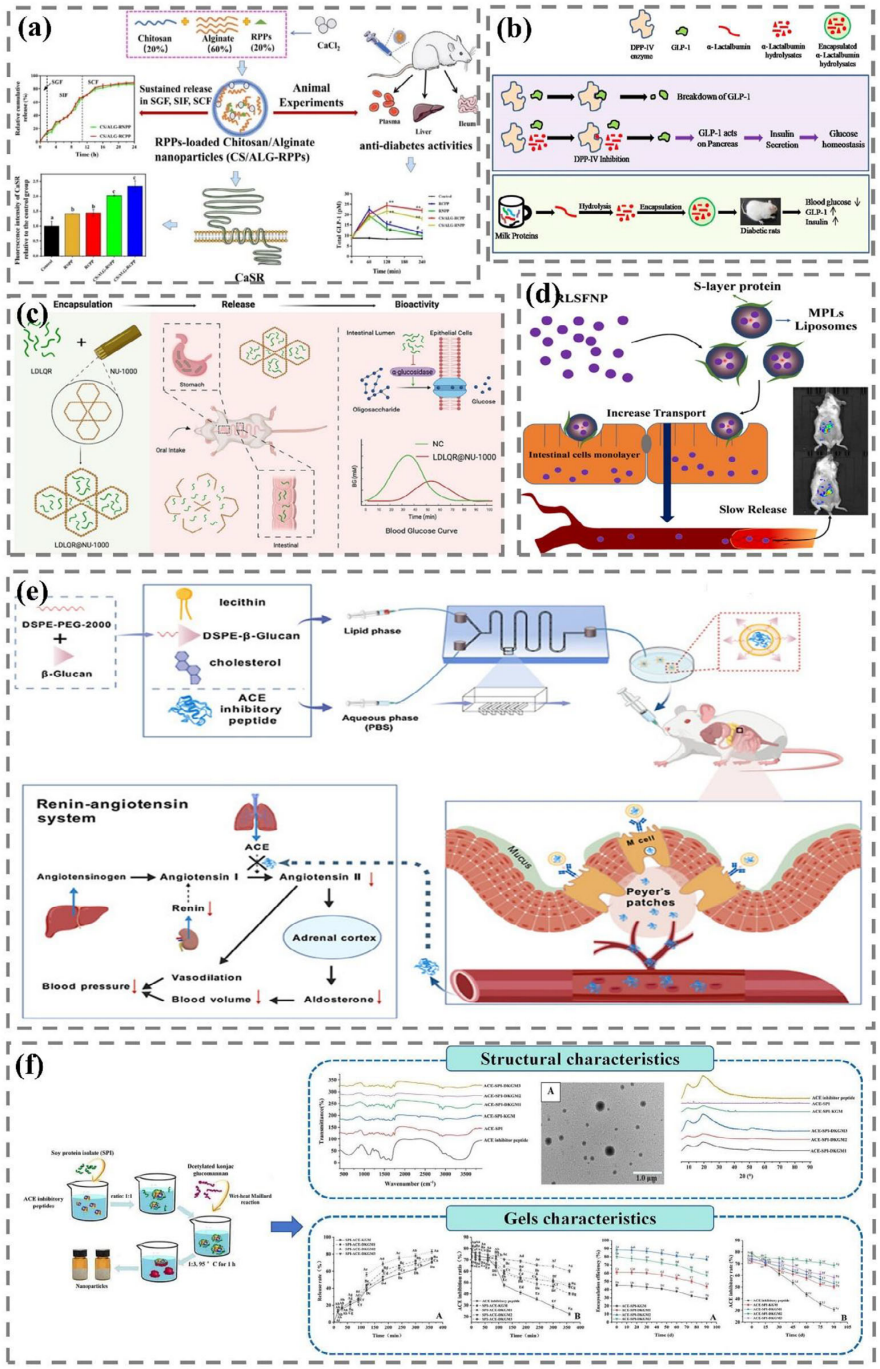
FBPs can effectively regulate metabolic diseases. (a) The chitosan (CS) and sodium alginate (ALG) composite delivery system delivers rapeseed peptides with hypoglycemic activity. The slowly released peptides can significantly improve glucose tolerance, increase the content of glucagon‐like peptide 1 (GLP‐1) in serum, and upregulate the expression level of CaSR. Reproduced with permission [128]. Copyright 2025, Elsevier Publishing Group. (b) The emulsified and encapsulated hydrolysate of α‐lactalbumin active peptide (EEH) reversed the abnormal metabolism of diabetic mice by reducing blood glucose levels and increasing GLP‐1 and insulin levels in plasma. Reproduced with permission [129]. Copyright 2023, American Chemical Society Publishing Group. (c) The acid‐resistant zirconium‐based metal‐organic framework NU‐1000 encapsulates wheat germ peptide Leu‐Asp‐Leu‐Gln‐Arg (LDLQR) to achieve intestinal targeted release and significant hypoglycemic effect. Reproduced with permission [130]. Copyright 2022, American Chemical Society Publishing Group. (d) The S‐layer protein‐modified liposome‐encapsulated natural ACE inhibitory peptide RLSFNP (Arg‐Leu‐Ser‐Phe‐Asn‐Pro), SLP‐LIP‐RLSFNP, improves the transmembrane transport efficiency of the peptide, promotes its absorption, and enhances its antihypertensive activity. Reproduced with permission [131]. Copyright 2021, American Chemical Society Publishing Group. (e) Egg white protein peptides (RADHPFL and YAEERYPIL) are precisely encapsulated in DSPE‐β‐glucan‐modified targeted liposomes, which effectively inhibit the production of angiotensin II, reduce organ damage, enhance metabolic regulation, repair endothelial dysfunction, and achieve an antihypertensive effect. Reproduced with permission [15]. Copyright 2025, American Chemical Society Publishing Group. (f) The pueraria mannose (DKGM) and soy protein isolate (SPI) delivery system encapsulates the ACE inhibitory peptide of jellyfish hydrolysate, ensuring the peptide activity while achieving stable and sustained release of the peptide. Reproduced with permission [132]. Copyright 2025, Elsevier Publishing Group.

Based on their core mechanisms of action, researchers have developed various targeted delivery strategies. Regarding glucose homeostasis regulation, constructing nanocarriers using natural polysaccharides represents a classic strategy for achieving sustained release and efficacy enhancement. Rapeseed peptides (RPPs) can precisely regulate human blood glucose homeostasis by inhibiting DPP‐IV activity and activating the calcium‐sensing receptor (CaSR). Wang et al. innovatively developed a stable nanocarrier system formed by electrostatic association of chitosan (CS) and sodium alginate (ALG) for encapsulating rapeseed‐derived cruciferous peptides (RCPP) and rapeseed peptides (RNPP), constructing CS/ALG‐RPPs and CS/ALG‐RNPP nanoparticles, respectively (Figure 12a). Both nanoparticles exhibited sustained‐release properties, with CS/ALG‐RPPs showing significant efficacy in improving metabolic indicators by substantially enhancing glucose tolerance, increasing serum glucagon‐like peptide 1 (GLP‐1) levels, and upregulating CaSR expression [128]. This study suggests that natural polysaccharide carriers not only provide gastrointestinal protection for peptides but may also mimic physiological secretion patterns through sustained‐release characteristics, thereby enhancing receptor agonist effects. However, batch‐to‐batch stability of chitosan/alginate systems and the heterogeneity of their degradation behavior in the complex intestinal environment remain concerns for translational applications.

Delivery efficiency optimization depends not only on carrier materials but also on the encapsulation form of peptides. Puri et al. investigated three different carrier forms for delivering α‐lactalbumin bioactive peptides: non‐encapsulated hydrolysate (NEH), freeze‐dried encapsulated hydrolysate (FDEH), and emulsified encapsulated hydrolysate (EEH) (Figure 12b). Among these, the EEH formulation demonstrated the best in vitro DPP‐IV inhibition (64 ± 2.02%). These hydrolysates successfully reversed metabolic abnormalities in diabetic model rats by reducing blood glucose levels and increasing plasma GLP‐1 and insulin levels. Furthermore, the lipid profiles, liver enzyme (ALT, AST, and AP) levels, as well as catalase and superoxide dismutase activities of experimental diabetic rats also trended toward normalization, achieving dual hypoglycemic and anti‐inflammatory effects [129]. The value of this study lies in revealing the decisive impact of delivery format on peptide functionality. However, it also suggests that excipient selection in emulsion systems and their interfacial interactions with peptides may potentially affect peptide conformational activity, requiring consideration in formulation design.

Furthermore, the introduction of structurally precise MOF materials can provide ultimate acid protection and targeted release for bioactive peptides. Liu et al. innovatively introduced the acid‐stable zirconium‐based metal‐organic framework NU‐1000 to encapsulate the wheat germ‐derived bioactive peptide Leu‐Asp‐Leu‐Gln‐Arg (LDLQR) (Figure 12c), which has been scientifically demonstrated to effectively inhibit α‐glucosidase activity. This carrier exhibited excellent encapsulation performance within just 10 min. The acid‐stable NU‐1000 ensured the integrity and stability of LDLQR and enabled precise intestinal release of LDLQR. In a mouse model of elevated blood glucose, LDLQR@NU‐1000 demonstrated significant hypoglycemic effects, proving that this carrier not only effectively delivers LDLQR but also possesses good biocompatibility without causing obvious inflammatory reactions or adverse effects on cell growth [130]. The breakthrough of this strategy lies in achieving spatial isolation protection of peptides through the pore confinement effect of MOFs. However, potential long‐term accumulation and in vivo degradation pathways require systematic evaluation.

Regarding blood pressure regulation and cardiovascular protection, delivery strategies focus on utilizing liposomes and nanoparticles to enhance gastrointestinal stability, targeted absorption capacity, and long‐acting sustained‐release performance of peptides. Zhang et al. prepared S‐layer protein‐modified liposomes encapsulating the naturally derived ACE inhibitory peptide RLSFNP (Arg‐Leu‐Ser‐Phe‐Asn‐Pro), forming SLP‐LIP‐RLSFNP (Figure 12d). During absorption, this system achieves precise recognition and targeted binding to target cells through specific interactions between S‐layer proteins and specific receptors on the cell surface, thereby substantially enhancing peptide transmembrane transport efficiency and promoting absorption. Simultaneously, it enables precise control of peptide release according to surrounding physiological conditions, achieving long‐acting and stable functional activity [131]. This biomimetic modification strategy endows liposomes with active targeting capability, but the matching relationship between modification density and receptor saturation, as well as potential effects on non‐target cells, requires further optimization.

Similar targeted modification strategies have also been applied to other liposome systems to comprehensively enhance delivery outcomes. Liu et al. precisely encapsulated egg white protein peptides (RADHPFL and YAEERYPIL) into DSPE‐β‐glucan‐modified targeted liposomes (Figure 12e). This system significantly improved peptide stability in the complex gastrointestinal environment while completely retaining their ACE inhibitory activity, ensuring potent biological functionality upon reaching target sites. Through fine regulation of the renin‐angiotensin system, egg white protein peptides effectively inhibited angiotensin II generation, thereby alleviating vasoconstriction and reducing blood pressure. Additionally, they significantly improved renal and cardiac functions, reduced organ damage, and enhanced the body's metabolic regulation capacity. Furthermore, they effectively repaired endothelial dysfunction, promoted vasodilation, and further contributed to stable blood pressure control [15]. This study demonstrates the dual advantages of targeted delivery and activity protection. However, the intestinal distribution characteristics of β‐glucan receptors and their potential effects on microbiota warrant further investigation.

Beyond liposomes, polysaccharide‐protein composite nanoparticle systems also provide reliable solutions for long‐term stable delivery. Cui et al. selected kudzu root glucomannan (DKGM) and soybean protein isolate (SPI) as core matrix materials to encapsulate ACE inhibitory peptides extracted from jellyfish hydrolysate (Figure 12f). This unique core‐shell structure not only effectively resists external environmental interference but also enables precise control of peptide release rates, achieving long‐acting and stable functional effects. During the 360‐min digestion process, the cumulative peptide release rate increased to 83.11%, while ACE inhibitory activity remained at 60.11%. These nanoparticles maintained good stability after 90 days of storage testing, with encapsulation efficiency retaining 76.56% of the initial value and ACE inhibitory activity maintaining 68.79%, demonstrating that this system significantly enhances the stability and sustained‐release performance of ACE inhibitory peptides while maintaining high activity after long‐term storage and simulated digestion [132]. This study provides a reference for room‐temperature storage and long‐acting formulation development of peptides. However, source variations in kudzu root glucomannan and its assembly mechanism with SPI require standardization research to ensure batch consistency.

In summary, through sophisticated delivery system design, FBPs can effectively overcome bioavailability limitations in metabolic disease intervention. However, it should be noted that some novel carriers, such as the zirconium‐based MOF NU‐1000 used for LDLQR delivery, lack sufficient data on in vivo degradation kinetics and potential organ accumulation, raising safety concerns that must be addressed before any practical application. Existing systems have achieved multi‐target intervention in glucose homeostasis maintenance, blood pressure regulation, and organ protection. Future research needs to further focus on long‐term carrier safety assessment, clinical translation pathway exploration, and the development of intelligent delivery systems with greater individualization potential, advancing this field toward precision nutrition and functional food applications.

### 5.4 Anti‐Cancer

FBPs exhibit distinct biological functions in regulating tumor cell cycles and remodeling the microenvironment. The occurrence and development of cancer involve multiple complex mechanisms, including gene mutation, metabolic reprogramming, immune escape, and tumor microenvironment imbalance. FBPs can exert their functions through various pathways, including directly inducing tumor cell apoptosis, regulating oxidative stress, inhibiting angiogenesis, and enhancing immune surveillance [133]. However, FBPs are prone to rapid clearance and degradation in vivo. Therefore, achieving activity protection, tumor site enrichment, and targeted regulation through nano‐delivery systems is crucial for fully realizing their anti‐tumor potential.

**FIGURE 13:**
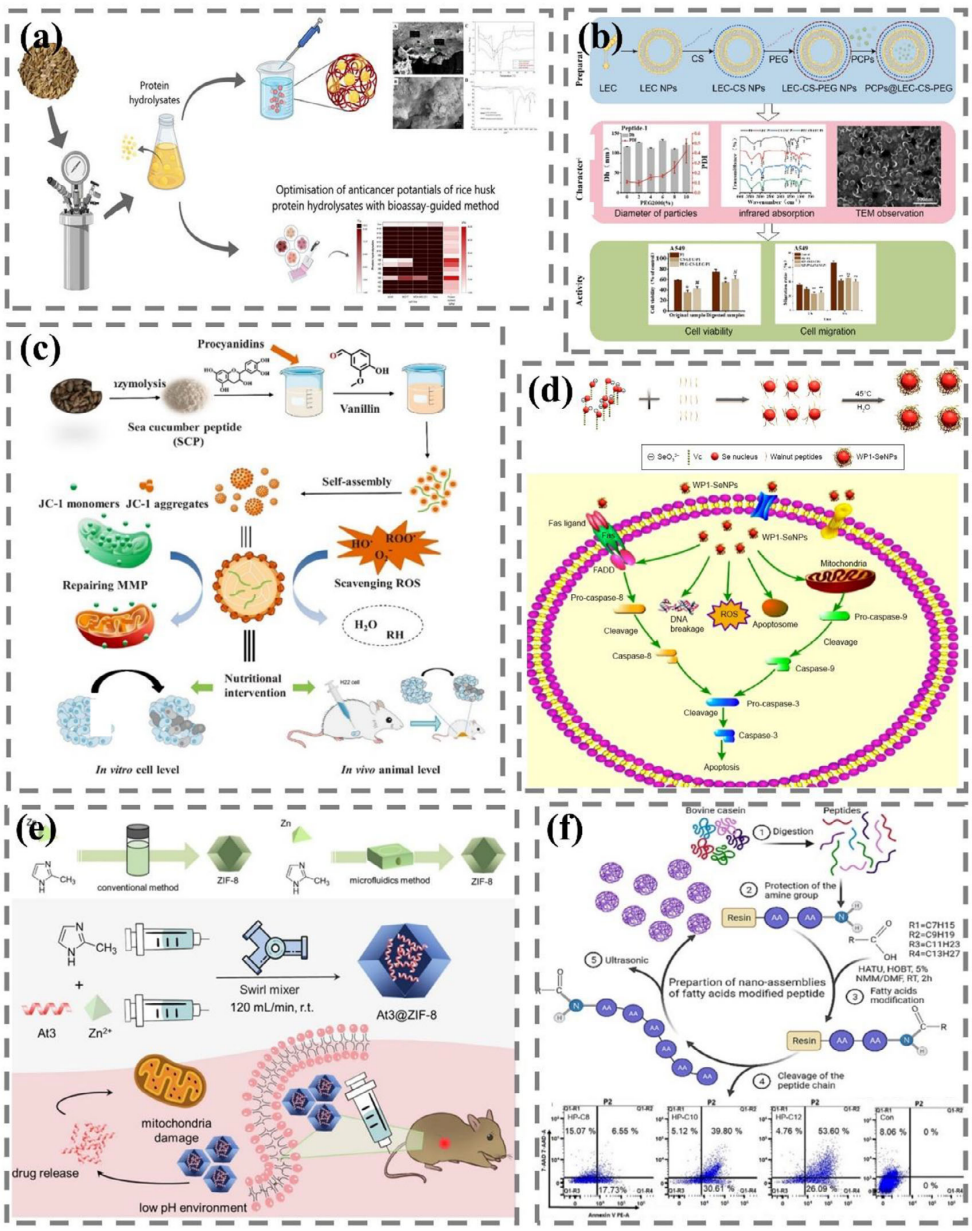
FBPs can effectively fight cancer. (a) Chitosan‐encapsulated rice husk peptides with anticancer activity exhibit strong inhibitory effects on A549 and MCF7 cancer cells with extremely low IC_50_. Reproduced with permission [134]. Copyright 2021, Elsevier Publishing Group. (b) Liposomes are used as the core and chitosan as the shell to encapsulate phycocyanin peptides (PCPs) with anticancer activity, which significantly enhances the inhibitory activity of PCPs against various NSCLC cells (including A549, H1299, and LTEP‐a2 cells). Reproduced with permission [104] Copyright 2025, MDPI Publishing Group. (c) The nanoparticle system constructed by sea cucumber peptide (SCP), proanthocyanidin (PC), and vanillin can effectively neutralize excessive ROS induced by H_2_O_2_ and acrylamide, stabilize mitochondrial membrane potential, and maintain the homeostasis of cellular energy metabolism, thereby significantly improving the survival rate of mice in liver cancer models and demonstrating its ability to enhance the anti‐tumor efficacy of SCP. Reproduced with permission [136]. Copyright 2023, Elsevier Publishing Group. (d) The selenium nanoparticle (WP1‐SeNPs) delivery system functionalized with defatted walnut peptide (WP1) can synergistically enhance its anticancer effect by regulating oxidative stress and energy metabolism. Reproduced with permission [137]. Copyright 2015, Taylor & Francis Publishing Group. (e) Zeolitic imidazolate framework‐8 (ZIF‐8) encapsulates the anticancer active ant‐derived peptide (At3). This system not only significantly reduces the hemolytic effect, but also significantly enhances cellular uptake, thus showing stronger anticancer efficacy. It can effectively inhibit the growth of multicellular tumor spheroids (MCTS) and destroy the mitochondrial membrane of MCF‐7 breast cancer cells. Reproduced with permission [138]. Copyright 2023, Elsevier Publishing Group. (f) Fatty acid‐loaded bovine casein hydrolyzed peptide (HP) significantly enhances the toxicity of the peptide to breast cancer cells and inhibits their migration and invasion. Reproduced with permission [139]. Copyright 2024, Elsevier Publishing Group.

Based on their core mechanisms of action, researchers have developed various delivery strategies. Regarding the induction of tumor cell apoptosis, encapsulating bioactive peptides using natural polysaccharide carriers represents a fundamental strategy to enhance their stability and cellular uptake. Ilhan‐Ayisigi et al. used chitosan as a nanocarrier to encapsulate rice husk peptides with anti‐cancer activity (Figure 13a). This delivery system exhibited potent inhibitory effects on A549 and MCF7 cancer cells, with extremely low IC50 values (1.98 and 3.58 µg/mL), attributed to the synergistic effects of diverse peptide/protein subunits with molecular weights ranging from 10 kDa to over 180 kDa in the rice husk protein hydrolysate [134]. This study suggests that synergistic effects among different components in natural peptide mixtures may surpass those of single peptides. However, component complexity and batch consistency pose challenges for mechanistic elucidation and quality control.

Regarding the suppression of tumor cell proliferation, constructing effective delivery systems is key to fully unleashing the anticancer potential of bioactive peptides, given their inherent instability. Taking phycocyanin peptides (PCPs) as an example, they can inhibit the growth and migration of non‐small cell lung cancer cells by targeting the epidermal growth factor receptor (EGFR) and inhibiting the downstream Akt signaling pathway [135]. However, their instability limits the realization of their anticancer potential. To address this, Cheng et al. constructed core‐shell nanoparticles (PCPs@LEC‐CS‐PEG) using lecithin (LEC), chitosan (CS), and polyethylene glycol (PEG) (Figure 13b). This delivery system significantly enhanced the tolerance of PCPs to heat, pH, and protease degradation. Furthermore, after nanoencapsulation, the inhibitory activity of PCPs against various lung cancer cell lines was markedly enhanced [104]. This strategy demonstrates the protective effect of carrier materials on peptide conformation. However, whether the cationic nature of chitosan may induce non‐specific interactions with biological barriers requires attention in subsequent studies.

In addition to directly triggering apoptosis programs, another type of strategy focuses on disturbing the redox and metabolic homeostasis of tumor cells to inhibit their survival. Huang et al. fused sea cucumber peptides (SCP), proanthocyanidins (PC), and vanillin to construct a nanoparticle system (Figure 13c). PC‐modified SCP nanoparticles effectively neutralized excessive reactive oxygen species (ROS) induced by H_2_O_2_ and acrylamide, while stabilizing mitochondrial membrane potential and maintaining cellular energy metabolic homeostasis, thereby significantly improving the survival rate of mice in a liver cancer model, demonstrating enhanced anti‐tumor efficacy of SCP [136]. This study illustrates the synergistic antioxidant effects between polyphenols and peptides. However, the spatiotemporal matching of their assembly ratio and release kinetics for synergistic effects requires further investigation.

Similarly, compounding bioactive peptides with inorganic nanoelements can produce synergistic enhancement effects. Ren et al. constructed a selenium nanoparticle (WP1‐SeNPs) delivery system functionalized with defatted walnut peptides (WP1) (Figure 13d). WP1‐SeNPs specifically inhibited the proliferation of cancer cells such as MCF‐7 by activating the Fas death receptor and caspase‐8 to initiate the exogenous apoptotic pathway; they also induced increased intracellular ROS levels and decreased mitochondrial membrane potential, thereby activating the endogenous apoptotic pathway, ultimately leading to cell cycle arrest, DNA fragmentation, and apoptotic execution [137]. These results demonstrate that this system synergistically enhances anti‐cancer effects by regulating oxidative stress and energy metabolism. However, the long‐term accumulation of selenium nanoparticles in vivo and their metabolic pathways require systematic evaluation.

To achieve more precise therapy and reduce side effects, intelligent responsive delivery systems have become an important development direction. Qiu et al. constructed zeolitic imidazolate framework‐8 (ZIF‐8) as a delivery carrier to encapsulate ant‐derived peptides (At3) with anti‐cancer activity, forming an At3@ZIF‐8 composite system that enables precise release in the slightly acidic tumor microenvironment (Figure 13e). This system not only significantly reduced hemolytic effects but also enhanced cellular uptake, thus exhibiting stronger anti‐cancer efficacy. It effectively inhibited the growth of multicellular tumor spheroids (MCTS) and damaged the mitochondrial membrane of MCF‐7 breast cancer cells [138]. The innovation of this strategy lies in utilizing the pH‐responsive properties of MOFs to achieve tumor microenvironment‐triggered precise release. However, the potential regulatory effects of ZIF‐8 degradation products on the tumor microenvironment, as well as their long‐term biocompatibility, require further investigation.

In addition, lipid‐based delivery systems can also effectively enhance the anti‐tumor activity of peptides. Song et al. used fatty acids as delivery carriers to precisely load bovine casein hydrolysate peptides (HP) for enhancing their anti‐breast cancer activity (Figure 13f). This system significantly enhanced their toxicity to breast cancer cells and inhibited their migration and invasion. In‐depth exploration of the apoptosis mechanism revealed that this may be closely related to the up‐regulation of the anti‐apoptotic protein Bcl‐2 and the pro‐apoptotic proteins Bax and caspase‐8 [139]. This study suggests that fatty acid carriers not only serve as delivery media but may also participate in lipid metabolism regulation, producing synergistic effects with peptides. However, the influence of fatty acid chain length and saturation on carrier performance and peptide function requires systematic optimization.

In summary, through the design and optimization of delivery systems, the anti‐tumor potential of FBPs can be exerted more safely and efficiently. Current research has made solid progress in carrier diversification, mechanism exploration, and preclinical model validation. However, a notable gap lies in the lack of selectivity assessment. Few studies include normal cell controls to determine whether the observed anti‐cancer effects are specific to cancer cells or also affect healthy cells. Without such data, the therapeutic window and potential off‐target toxicity of these delivery systems remain unclear. Future challenges lie in: first, deepening the understanding of dynamic interactions between carriers and the tumor microenvironment, particularly the potential regulatory effects of carrier materials on the tumor immune microenvironment; second, systematically conducting clinical translation evaluations, including scalable preparation, quality control, and long‐term safety studies; and third, promoting the development of intelligent delivery platforms capable of adapting to tumor heterogeneity and dynamic microenvironmental changes to achieve individualized precision intervention.

### 5.5 Others

FBPs exhibit distinct biological functions in maintaining immune homeostasis and regulating bone metabolism. Immune imbalance can lead to the formation of a chronic inflammatory microenvironment and impaired immune surveillance, while bone metabolic diseases such as osteoporosis are closely associated with disruption of osteoblast‐osteoclast homeostasis [140]. FBPs exert multi‐target regulatory effects through activating immune cells and modulating key signaling pathways such as Wnt/β‐catenin. However, rapid in vivo degradation and insufficient target site accumulation limit their functional efficacy. Nano‐delivery systems can significantly enhance their stability and bioavailability through efficient encapsulation and precise delivery, achieving the dual goals of efficacy enhancement and reduced systemic toxicity.

**FIGURE 14:**
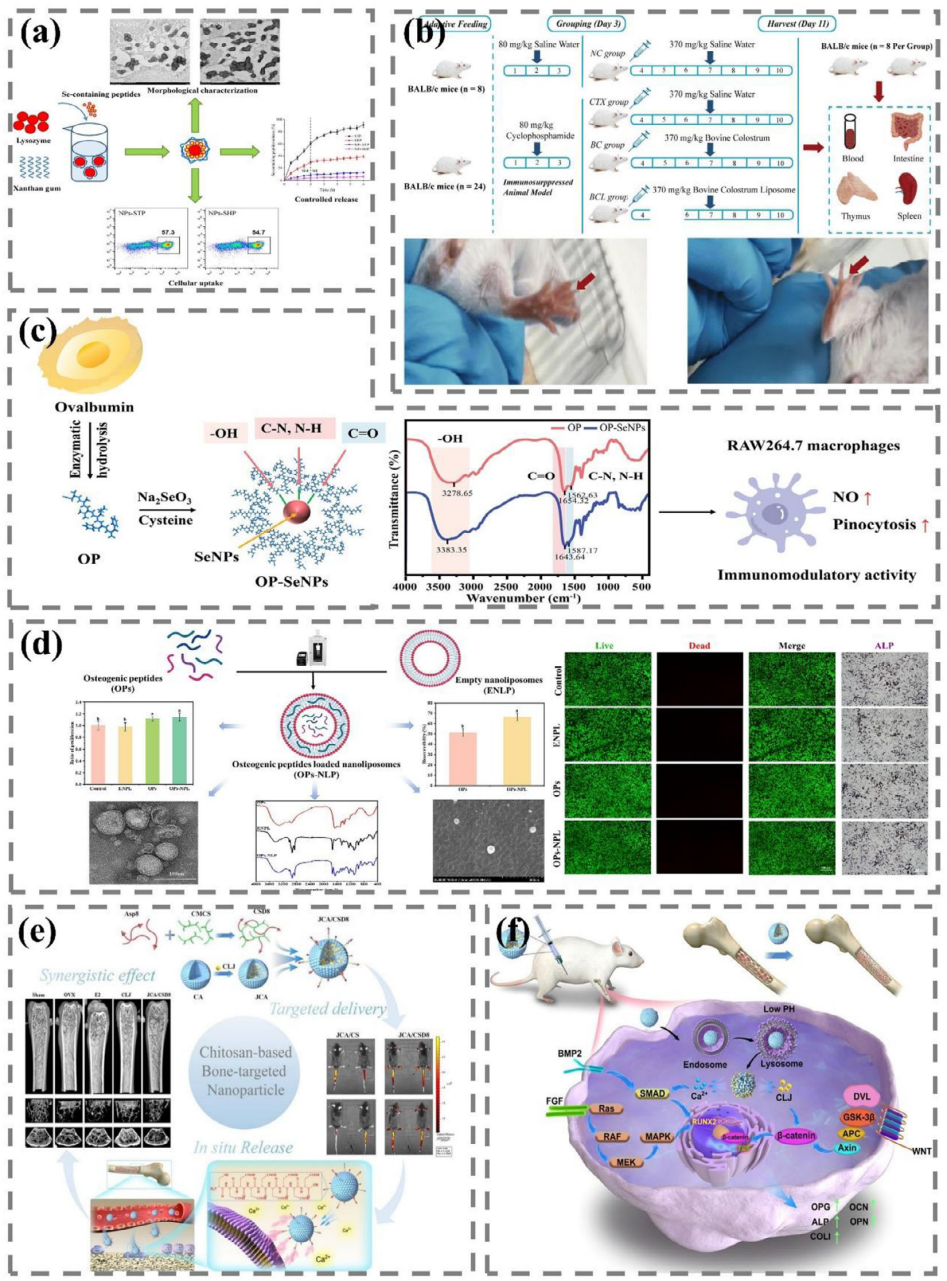
FBPs used for the regulation of other diseases. (a) Nanoparticles (NPs) co‐constructed by xanthan gum and lysozyme loaded with selenium‐containing immunologically active peptides, which greatly enhance the transmembrane transport capacity of selenium‐containing peptides. Reproduced with permission [141]. Copyright 2022, Elsevier Publishing Group. (b) Liposomes encapsulating bovine colostrum immunoglobulin can effectively restore cytokine levels and organ function under immunosuppressive conditions. Reproduced with permission [142]. Copyright 2024, Royal Society of Chemistry Publishing Group. (c) The OP‐SeNPs delivery system constructed by selenium (Se) and ovalbumin peptides (OP) can enhance pinocytosis activity to promote cellular uptake by activating macrophages and promoting nitric oxide secretion, further enhancing the innate immune response. Reproduced with permission [143]. Copyright 2025, Elsevier Publishing Group. (d) Liposome (NLP) encapsulation of osteogenically active cod bone (OPs) significantly improves its bioavailability. Reproduced with permission [144]. Copyright 2024, Elsevier Publishing Group. (e) A novel nanoparticle (JCA/CSD8) is obtained by modifying the surface of nanoparticles loaded with flaxseed cyclopeptide J (CLJ) with a bone‐targeting polymer conjugate (CSD8) prepared from carboxymethyl chitosan (CMCS) and functional peptide (ASP8), which can significantly improve the affinity with bone tissue and enhance the improvement of osteoporosis. Reproduced with permission [145]. Copyright 2025, Elsevier Publishing Group. (f) The nanocomposite system (JCA) of porous calcium carbonate (CA) loaded with flaxseed cyclopeptide J (CLJ) enables efficient targeted delivery to enhance bone formation. Reproduced with permission [146]. Copyright 2023, Elsevier Publishing Group.

In the context of immune regulation, delivery systems are primarily dedicated to enhancing peptide stability and cellular uptake efficiency. Zhu et al. constructed nanoparticles (NPs) using xanthan gum and lysozyme to encapsulate selenium‐containing bioactive peptides TSeMMM (STP) and SeMDPGQQ (SHP) (Figure 14a). Following encapsulation, the stability and antioxidant activity of these selenium‐containing peptides were significantly improved. Furthermore, this carrier exhibited extremely low toxicity and efficiently entered Caco‐2 cells via clathrin‐mediated endocytosis, substantially enhancing the transmembrane transport capacity of selenium‐containing peptides [141]. This study suggests that polysaccharide‐protein composite carriers not only provide physical protection but also that their surface characteristics can regulate cellular uptake pathways. However, the influence of the assembly ratio between xanthan gum and lysozyme on nanoparticle surface charge and mucus penetration, as well as their disassembly behavior in the complex intestinal environment, requires systematic optimization.

Building upon this foundation, optimizing absorption efficiency is critical for enhancing overall immunomodulatory function. Zhou et al. prepared liposome‐encapsulated bovine colostrum immunoglobulin, which significantly improved absorption efficiency in a mouse model (Figure 14b) and effectively restored cytokine levels and organ functions under immunosuppressive conditions [142]. This study demonstrates the protective advantages of liposomes for protein‐based bioactive substances. However, their gastrointestinal stability and transepithelial transport mechanisms require further elucidation. The influence of liposomal phospholipid composition and particle size on endocytic pathways in intestinal epithelial cells and subsequent intracellular trafficking fate warrants further investigation.

Beyond passive enhancement of delivery efficiency, actively activating immune cell responses represents a more advanced strategy. Zeng et al. constructed an OP‐Se NPs delivery system using selenium (Se) and ovalbumin peptides (OP) (Figure 14c). Se NPs exhibited positive interactions with ‐OH, ‐CO, ‐CN, and ‐NH groups in OP molecules, ensuring tight combination and stable delivery of Se and OP. Furthermore, OP‐Se NPs enhanced innate immune responses by activating macrophages, promoting nitric oxide secretion, and enhancing macropinocytosis activity to facilitate cellular uptake [143]. The innovation of this strategy lies in integrating the immunomodulatory function of inorganic elements with the targeting recognition properties of peptides, achieving “carrier‐active ingredient” bifunctional synergy. However, the long‐term in vivo accumulation of selenium nanoparticles and their potential regulatory effects on the immune system require long‐term safety assessment.

Regarding bone metabolism regulation, delivery system design emphasizes achieving bone tissue targeting and microenvironment modulation. Zhu et al. used nanoliposomes (NLP) to encapsulate cod bone peptides (OPs) (Figure 14d). The close combination of OPs with blank NLP through hydrogen bonding and hydrophobic interactions not only improved OP stability but also enabled sustained in vivo release without affecting their activity, thereby improving their bioaccessibility [144]. This study provides a feasible approach for oral delivery of bone peptides. However, liposomes lack active bone‐targeting capability, potentially leading to peptide distribution in non‐target organs and increased systemic exposure risk.

To achieve more precise bone tissue enrichment, active targeting modification of carriers represents an effective approach. Chen et al. prepared bone‐targeted polymer conjugates (CSD8) using carboxylated chitosan (CMCS) and functional peptides (ASP8). Subsequently, CSD8 was modified onto the surface of nanoparticles loaded with flaxseed cyclopeptide J (CLJ), thus developing a novel nanoparticle system JCA/CSD8 (Figure 14e). JCA/CSD8 significantly improved affinity for bone tissue, enabling precise release of active ingredients and calcium ions in the acidic lysosomal environment while synergistically activating osteogenesis‐related pathways, thereby effectively increasing bone mineral density and repairing trabecular bone structure in an osteoporosis mouse model [145]. The breakthrough of this study lies in achieving dual functionality of bone targeting and microenvironment‐responsive release. However, the bone‐binding specificity of ASP8 and its potential recognition of other bone matrix components, as well as the optimal pH matching between the acidic release environment and osteoblast function, require further investigation.

Furthermore, carrier materials themselves can actively participate in synergistic regulation of osteogenic signals. Chen et al. previously selected porous calcium carbonate (CA) nanoparticles to construct a nanocomposite system, JCA, also loaded with CLJ (Figure 14f). This system not only ensured stable CLJ loading but also endowed it with efficient delivery to target sites. CLJ and CA synergistically regulated two key signaling pathways, Wnt/β‐catenin and BMP/Smad, activating a series of osteogenic factors, thereby comprehensively promoting bone formation processes [146]. This strategy represents a paradigm shift from “passive delivery” to “active synergy” of carrier materials. However, the matching between calcium carbonate degradation rate and CLJ release kinetics, as well as the potential regulatory effects of locally elevated calcium concentrations on the bone microenvironment, require systematic optimization and evaluation.

In summary, through intelligent design of delivery systems, FBPs can achieve more efficient and precise functional intervention in the fields of immune regulation and bone metabolism. Existing research has confirmed the feasibility of carrier construction, targeted delivery, and mechanistic synergy. Future studies need to further explore the long‐term in vivo fate of carriers, clinical translation pathways, and individualized adaptation strategies. In‐depth investigation of the synergistic regulatory mechanisms between carrier materials and FBPs within the immune microenvironment and bone metabolic network will advance this field toward precision nutrition and functional food applications.

## 6 Conclusions and prospects

FBPs offer a novel and relatively safe strategy for chronic disease management by virtue of their biological functions, including antioxidant, anti‐inflammatory, and glycemic regulatory activities. However, the inherent challenge of their low oral bioavailability severely restricts their clinical translation from nutritional supplements to functional therapeutic preparations.

Although the diversified development of current delivery systems has improved peptide stability to some extent, there remains a general lack of precise understanding and regulation of their in vivo delivery fate. The design of nanocarriers and composite systems is predominantly based on empirical optimization, with superficial understanding of trans‐mucosal transport mechanisms, carrier metabolic pathways, and their long‐term biocompatibility, resulting in insufficient predictability and reproducibility of delivery efficiency. For instance, while existing studies have demonstrated that chitosan nanoparticles can enhance peptide transepithelial transport, systematic quantitative analyses are lacking regarding their diffusion kinetics within the mucus layer, specific recognition mechanisms with intestinal epithelial cell surface receptors, and the extent of conformational activity retention of peptides following carrier disassembly. This “black box” understanding of the delivery process limits rational and precise carrier design.

Future breakthroughs lie in moving beyond simple physical encapsulation toward the rational design of intelligent responsive delivery systems that seamlessly merge processing and environmental stability factors – such as metal ions, enzymes, and pH – with strategies to overcome oral absorption barriers. Metal ions, for example, can be harnessed to enhance peptide stability through coordination‐driven conformational rigidification, preventing aggregation and enzymatic degradation during both manufacturing and gastrointestinal transit. Enzymes and pH fluctuations, while inherently threatening to peptide activity, can be deliberately exploited as biochemical triggers to construct enzyme‐ or pH‐responsive carriers. These carriers are designed to shield the peptide in hostile compartments (e.g., the strongly acidic and pepsin‐rich stomach) and release it precisely at intestinal absorption sites, thereby converting potential destructive factors into site‐specific delivery signals. This necessitates deep integration of materials science with intestinal physiology to develop carriers capable of precisely sensing gastrointestinal microenvironmental cues (such as pH gradients, enzyme distribution profiles, and redox potential) and triggering controlled release. More importantly, elucidating the molecular mechanisms underlying dynamic interactions among carriers, peptides, and biological barriers is imperative, including structure‐activity relationships between carrier surface physicochemical properties and mucus penetration, selective activation mechanisms of transcellular transport pathways, and the potential regulatory effects of carrier materials on gut microbial ecology, thereby providing theoretical guidance for efficient delivery.

The core challenge for achieving clinical translation lies in establishing a standardized bridge connecting fundamental research and industrial application. It is essential to promote systematic investigations covering carrier pharmacokinetics, long‐term safety, and scalable manufacturing processes, while actively exploring the interaction network between FBPs and the gut microbiota. For example, do degradation products of carrier materials possess potential immunogenicity or cytotoxicity? Does peptide release in the colon selectively modulate the metabolic activity of specific microbial populations, thereby generating indirect physiological effects? Addressing these questions will provide critical support for unlocking the full potential of FBPs in the systemic treatment of chronic diseases such as metabolic disorders.

Ultimately, the development of FBPs will undergo a paradigm shift from fragmented functional verification toward the construction of delivery platforms based on precision medicine demands. By integrating computational simulation‐assisted carrier design, personalized formulation optimization, and rigorous clinical functional evaluation, this field is poised to generate a new generation of intelligent functional foods and therapeutic preparations for chronic disease management.

## Author Contributions

**Yu Xu**: conceptualization, methodology, investigation, supervision, funding acquisition, project administration, resources, Writing – original draft, Writing – review and editing, data curation. **Tao Shu**: validation, visualization. **Weihong Min**: supervision, resources, writing – review and editing. **Bei‐Wei Zhu**: supervision, writing – review and editing, resources, conceptualization. **Runan Zhao**: writing – review and editing, methodology, resources. **Fan Li**: methodology, software, data curation, investigation, validation, formal analysis, visualization, writing – original draft.

## Conflicts of Interest

The authors declare no conflicts of interest.

## Data Availability

The data sharing is not applicable to this article as no datasets were generated or analyzed during the current study.
